# Cognition and future depression: associations with risk in those with and without a history of depression

**DOI:** 10.1136/bmjment-2025-302332

**Published:** 2026-05-07

**Authors:** Angharad N de Cates, Angeline Lee, Laura Winchester, Klaus P Ebmeier, Paris Lalousis, Rachel Upthegrove, Susannah E Murphy, Catherine J Harmer, Thomas Nichols, Anya Topiwala

**Affiliations:** 1 Institute for Mental Health, University of Birmingham, Birmingham, UK; 2 Department of Psychiatry, University of Oxford, Oxford, UK; 3 Big Data Institute, University of Oxford, Oxford, UK; 4 Oxford Health NHS Foundation Trust, Oxford, UK; 5 Institute of Psychiatry Psychology & Neuroscience, King’s College London, London, UK

**Keywords:** Depressive Disorder, Cognition Disorders, Psychological Tests

## Abstract

**Background:**

Cognitive impairments are common in depression and often persist beyond mood resolution. However, the relationship between cognitive performance, its neurological underpinnings, and future depression risk is unclear, limiting strategies for primary and secondary prevention.

**Objective:**

Our objective was to determine whether cognition associates with subsequent depression, both relapse and first-episode occurrences.

**Methods:**

1862 UK Biobank participants with a history of International Classification of Diseases (ICD)-10-defined depression in remission (RD) (mean (SD) age: 52.7 (7.13) years) were age-matched and sex-matched to 1862 participants without depression history or current antidepressant use. Cognitive scores were compared between groups at the composite (z-score), domain and task levels. MRI-derived phenotypes assessed brain network structure and functional connectivity. Longitudinal associations with future depression were assessed using logistic regression models and a Cox proportional hazards model controlling for key confounders.

**Findings:**

Participants with RD had a higher risk of future depression (33%) than controls (13%), including when we accounted for temporal differences in longitudinal assessment (HR=3.16 (95% CI 2.71 to 3.67), global proportional hazard assumption p=0.07). Composite cognitive performance in controls was inversely associated with future depression risk (risk estimated marginal means: 0.25% at −1SD, 0.20% at mean, 0.15% at +1 SD). In RD, this relationship was reversed (0.74% at −1SD, 0.90% at mean, 1.10% at +1 SD). Executive functioning, processing speed and reasoning task scores all contributed. Higher grey matter in default mode network regions was associated with better concurrent cognitive performance across all participants, but not with future depression risk. Other MRI findings were limited.

**Conclusions:**

RD carried a threefold higher risk of future depression than controls. Cognitive performance was a risk marker for future depression in both groups but in opposing directions. Neuroimaging metrics provided little predictive value.

**Clinical implications:**

Personalised risk factor assessment for depression is likely to be dependent on depression history. Those without previous history of diagnosed depression are at higher risk of future depression when cognitive performance is lower at baseline. RD is a high-risk group for future depression, and those with relatively higher cognitive performance may be more likely to report future depressive symptoms.

**WHAT IS ALREADY KNOWN ON THIS TOPIC**

- Neurocognitive impairments are common in depression, even after low mood has resolved and outside of comorbid neurodegenerative processes. However, the specific relationship between cognitive performance and risk of future depression is unclear, including how this relates to previous history of depression.

**WHAT THIS STUDY ADDS**

- Cognitive performance is a differential risk marker for future depression in those with previous depression compared with matched controls: without previous history of depression, poorer cognitive scores confer the highest risk; in remitted depression, higher levels of cognitive performance are associated with greater risk of depressive relapse.

**HOW THIS STUDY MIGHT AFFECT RESEARCH, PRACTICE OR POLICY**

- Further research should explore targeting interventions based on specific cognitive profiles, especially in high-risk populations such as those with previous episodes of depression.

## Background

 Major depressive disorder affects one in five people in their lifetime with significant morbidity and mortality,^1^ and poor cognition is a main component of the diagnosis for all ages, affecting 70%–90% of those affected.^2 3^ Individuals with a history of depression appear to face a significantly elevated risk of relapse,^1^ but the ability to predict which individuals are most susceptible remains limited—despite its potential to inform targeted prevention and improve clinical outcomes. Cognitive impairments persist in approximately 40% of patients (including deficits in attention and executive functioning, processing speed and learning and memory), even after mood symptoms remit^4^,^6^ and respond poorly to first-line antidepressants.^7^,^9^ Cognitive problems also tend to worsen with successive episodes,^4 5 10^ suggesting that they may hold promise as a robust prospective marker of risk for depressive relapse. However, despite the potential clinical importance, longitudinal evidence directly linking cognitive deficits to future depression risk is currently lacking, including details of which cognitive domains may be most relevant for relapse and therefore a potential target for preventative measures.

A proposed mechanistic link between poor cognition and depressive relapse is dysconnectivity within the default mode network (DMN), central executive network (cEN) and salience brain network (SN) (also known as the ‘triple network’.^11^ Alterations in the triple network are noted in both current and remitted depression (RD) compared with controls,^12^,^14^ and these networks also directly link to cognitive performance (ie, worse cognition is associated with weaker DMN-SN and SN-cEN connections).^15^ This suggests both impaired cognition and network connectivity may mark relapse risk,^16 17^ but uncertainty remains as to how the structural and functional integrity of these networks might interact with cognition to predict future depressive episodes.

## Objective

Using UK Biobank (UKB) data, we conducted a large, prospective, multimodal study aiming to identify cognitive and neural markers of depression onset and relapse vulnerability. We also assessed whether structural and functional properties of the triple network mediate these associations. We hypothesised that poorer cognition would predict future depression, particularly in participants with RD (primary analyses), and that abnormalities within the triple network and constituent brain regions would contribute to this risk (secondary analyses).

## Methods

### Study sample

**Figure 1:**
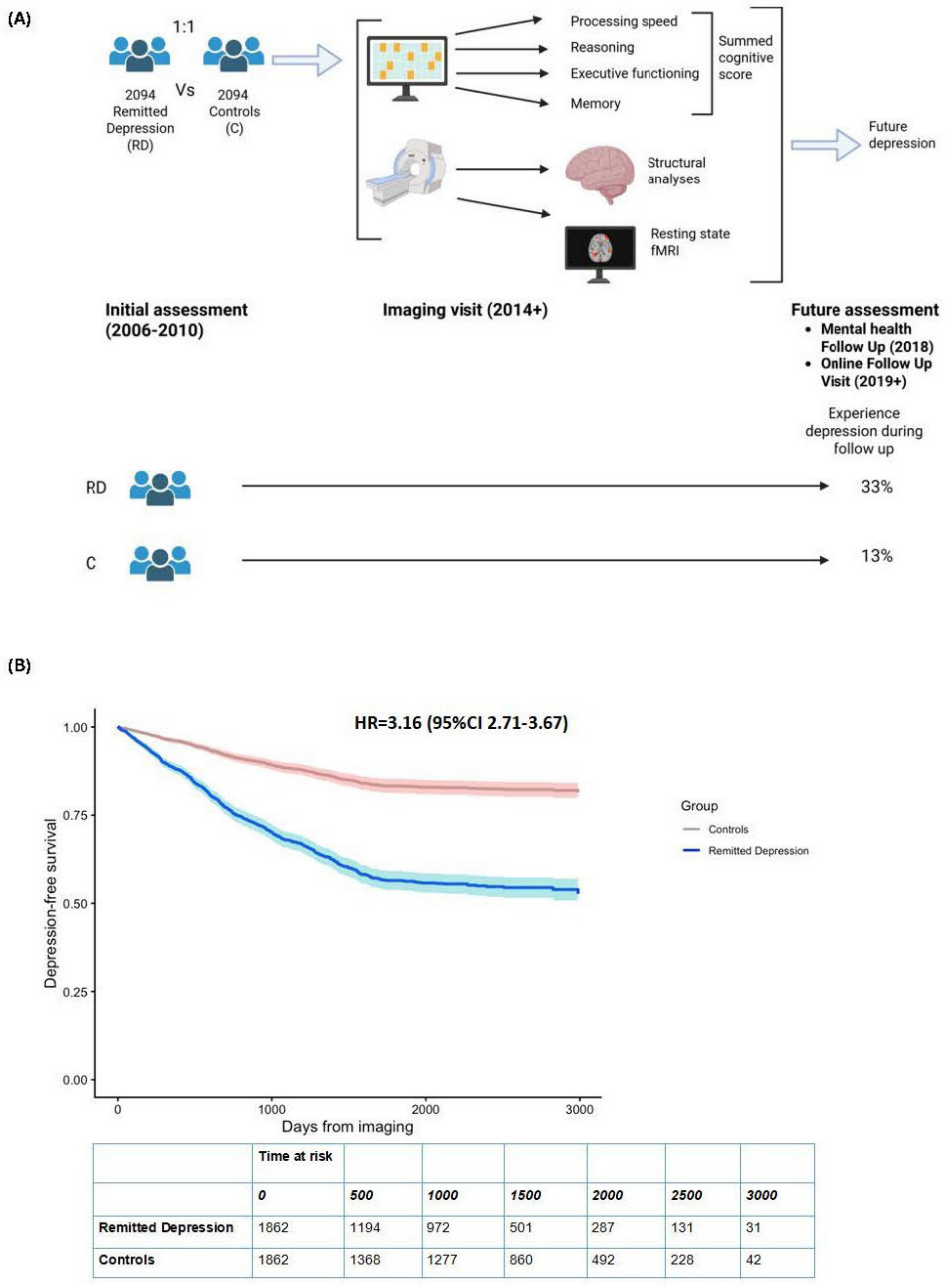
(**A**) Created in Biorender. (**B**) Kaplan-Meier curves showing the cumulative incidence of depression-free survival over follow-up in remitted depression vs matched control participants (max 2988 days for those developing depression; max 3249 days for no development of depression). The shaded areas around curves represent 95% CI. n=3724; number of events=863: 623 in RD cohort and 240 in control cohort. Number censored due to death across both cohorts=50. Global proportional hazards assumption, p=0.07. Included table shows the numbers of participants at risk over time for each group (remitted depression vs controls). fMRI, functional MRI; RD, remitted depression.

An overview of methodology is shown in figure 1. The study comprised participants from the UKB as outlined in the online supplemental material (online supplemental figure 1; see completed Strengthening the Reporting of Observational Studies in Epidemiology statement). The UKB is a prospective cohort study involving approximately 500 000 participants from the UK. These individuals were aged 40–69 years at the time of their initial assessment visit, conducted between 2006 and 2010. Participants provided informed consent via electronic signature at the time of recruitment. Additional details regarding the UKB protocol can be accessed through their website (http://www.ukbiobank.ac.uk/). In brief, invitations to join the Biobank were sent to people registered with NHS general practices near up to 35 assessment centres across England, Wales and Scotland. Participation was voluntary, and people with existing illnesses could take part. The primary aim was to create a prospective resource to investigate how genetic, lifestyle and environmental factors jointly influence risk of common diseases of middle and older age with long-term follow-up through linkage to National Health Service (NHS) records.

Sociodemographic data used in this study were from the initial assessment visit (2006–2010) and the first imaging visit (2014–2019). Baseline cognitive measures and MRI data used in the study were collected during the first imaging visit.

RD was defined as (1) a recorded depressive episode occurring up to 15 years before the first imaging visit, and (2) remission from depressive symptoms at the first imaging visit defined as scoring 1 (not at all) or 2 (several days but less than half the days) on each of the two self-report Patient Health Questionnaire (PHQ)-2 core questions (self-reported frequency of low mood and anhedonia symptoms in the last 2 weeks; PHQ-9 was not available at the imaging visit). As patients with depression usually self-identify higher scores on measures of depressive severity than treating clinicians,^18^ patient-reported remission is likely to be of clinical validity. For the primary cohort, an International Classification of Diseases (ICD)-10 diagnosis of at least moderate severity depression using first occurrence and hospital episode statistics (FO+HES) data was used for (1) (see online supplemental material, pp. 6–8). We also conducted sensitivity analyses altering the threshold for depressive episode using: (a) general practice (GP) ICD codes for moderate to severe ICD-10 depression for those with linked primary care records and (b) the ‘probable depression’ UKB-derived algorithm derived from self-report variables.^19^ Details of codes used in cohort definitions and an overview of cohort inclusion criteria are available in online supplemental data, pp. 5–9.

Cohort participants with RD with confounder, cognitive and functional MRI (fMRI) data were matched 1:1 with control participants (with no history of depression) drawn from the remainder of the UKB based on availability of required data, sex and age +/−2 years, and no lifetime history of ICD-10 coded depression (as defined by inclusion in the FO+HES cohort or the GP linked data cohort), or inclusion within the self-report ‘Probable Depression’ cohort, or antidepressant use at the imaging visit (using tabular data antidepressant codes). We also excluded from all RD and control cohorts those with current significant neurological/neurodegenerative disorders, or development of these within the following 2 years (see online supplemental methods). Other mental disorders were included as potential confounders.

### MRI acquisition and data processing

The imaging data were obtained on Siemens Skyra 3T MRI scanners equipped with 32-channel head coils. The UKB team performed image processing, quality control checks and automated brain tissue volume computations; their imaging-derived phenotypes were made available to the researchers. The brain imaging protocol used in the UKB includes structural, diffusion and functional imaging from six distinct modalities: T1-weighted, T2-weighted flair, diffusion MRI, susceptibility-weighted imaging, task fMRI and resting-state functional MRI (rsfMRI) time series data.^20^

Previous UKB studies^15 21^ suggested networks of interest for cognition in depression include the following 11 components (nodes) based on the triple network of the DMN, cEN/frontoparietal network (FPN), and SN: (1) DMN=1, 7, 9, 14, 20; (2) cEN/FPN=5, 6, 16, 21 (5, 6 and 21 are part DMN, part cEN) and (3) SN=3, 13 (13 is part DMN, part SN). 54 edges involving these 11 nodes were taken from https://www.fmrib.ox.ac.uk/datasets/ukbiobank/group_means/edge_list_d25.txt. T1-weighted measures estimated grey matter and cortical measures. Structural regions of the triple network were pre-specified as amygdala, anterior cingulate cortex, hippocampus, medial frontal gyrus, precuneus, posterior cingulate cortex, supramarginal gyrus and superior frontal gyrus (bilaterally). rsfMRI was conducted at two distinct dimensionalities (25 and 100), resulting in 21 and 55 signal networks, providing information on measures of both within-network and between-network functional connectivity. Additional details on the acquisition parameters, image processing and specific measurements derived from both imaging modalities are available in online supplemental methods.

### Details of cognitive assessment

Cognitive behavioural tasks were performed at the imaging visit. Individual tasks were defined by the cognitive domain being tested: (1) Processing speed=Reaction Time [‘Snap-card game’] (UKB_ID_20023: mean time to correctly identify matches from eight trials) and Symbol-Digit Substitution (UKB_ID_23324: number of symbol digit matches made correctly); (2) Reasoning=Verbal-Numerical Reasoning [‘Fluid Intelligence’] (UKB_ID_10016: sum of correct answers/fluid intelligence score) and Matrix Pattern Completion (UKB_ID_6373: number of puzzles correctly solved); (3) Attention and executive function=Digit Span [‘Numeric Memory’] (UKB_ID_4282: maximum digits recalled), Trails A and B (UKB_ID_6350: duration to complete alphanumeric path #2 MINUS 6348: duration to complete numeric path #1), Tower Rearranging (number of puzzles correct) and (4) Memory=Verbal Paired Associates (UKB_ID_20197: number of word pairs correctly associated). The psychometric properties of these tasks have previously been reported.^21^

Calculation of a composite cognitive score was based on previous methodology,^22^ that is, by calculating a composite z-score using the mean scores for each task. Separate z-scores were also created for each cognitive domain and each individual task. Participants with incomplete data were excluded from the main composite cognitive analyses. However, if data from at least three tasks were available, missing values were imputed, and these participants were included in a sensitivity analysis (see Statistical analyses section). For analyses of individual cognitive domains and tasks, participants were included when data were available.

### Longitudinal outcome data

A depressive episode occurring in either the RD cohorts or matched cohorts after the imaging visit for participants with suitable data was identified using one of the following (see online supplemental methods, p. 5 for details): (1) presence of an HES depression code after the imaging time point (data available up to 31 March 2023) or (2) a PHQ-9 score greater than 9 on mental health follow-up (MHFU) data (collected from 2019) if the imaging visit occurred before MHFU or (3) MHFU age at the last episode of depression if greater than age at the imaging visit (collected 2016–2017) or (4) recent depression recorded at the online follow-up visit (collected from 2019) after imaging. Only participants with longitudinal data available for at least 2 months after their imaging visit were included in analyses.

Models for both primary (cognitive) and secondary (neural/neurocognitive) analyses were pre-specified and are listed in online supplemental methods (p. 9). We adjusted for potential confounders, which were self-reported or established at the time of the MRI scan (apart from sex, ethnicity, deprivation score and education, which were assessed at the initial assessment visit). A full list of these is given in the online supplemental material. In brief, Townsend deprivation is a measure of material deprivation based on address census information. Educational qualifications were recorded as: college or university degree, A level/AS levels or equivalent, O levels/GCSEs or equivalent, Certificate of Secondary Education (CSEs) or equivalent, National Vocational Qualification (NVQ) or Higher National Diploma/Certificate (HND or HNC) or equivalent, other professional qualifications or none of the above. Smoking was reported as: current, previous or never. Alcohol status was as units consumed per week. Body mass index (BMI) in kg/m^2^. For MRI analyses, age^2^, age^3^ and age-by-sex interaction were accounted for as well as a set of brain imaging-related confounders (see online supplemental file 1).

### Statistical analyses

All statistical analysis was performed in RStudio (V.4.4.1). Independent-samples t-tests and χ^2^ tests were performed to assess potential univariate differences in the sociodemographic characteristics between participants with RD and controls. Binomial logistic regression was performed for both primary (cognitive) and secondary (neural/neurocognitive) analyses, accounting for confounders as detailed above. Where appropriate, regression models were performed with and without group (ie, RD or control) as a factor to identify relationships across both cohorts. P values in secondary analyses were corrected for multiple testing using false discovery rate (5%).

As well as repeating primary analyses with alternative cohorts as previously described (GP or the ‘Probable Depression’ algorithm data), we performed other sensitivity analyses as follows: (1) extending the exclusion of participants who developed neurodegenerative disease or dementia from 2 to within 10 years after imaging; (2) excluding participants who had cerebrovascular disease at imaging and (3) controlling for participants with a close family history of depression. We also repeated analyses including a marker of socioeconomic status (Townsend Deprivation Index (TDI)) as an interaction term in the analysis model rather than a covariate, after dividing the primary (FO+HES) cohort into participants up to 64 and those over 64 years old, and using a less stringent criteria for controls (not in the FO+HES RD cohort and PHQ-2 scores <3 for each question at imaging). Imputation of missing cognitive data was used as a sensitivity analysis. Prior checks ensured that data were missing at random to confirm the validity of imputation (ie, individual cognitive task scores, age and sex were not statistically different in those with and without full cognitive data). The R package MICE was used to impute cognitive data, using predictive mean matching to preserve relationships between variables, and with age and sex included as auxiliary variables using 20 imputations (see online supplemental material for details). Estimated marginal means were calculated using the emmeans R package. A multivariable Cox proportional hazards regression model was fitted to estimate adjusted HRs and 95% CIs for the association between group status (RD vs controls) and time to incident depression, defined as the first recorded diagnosis of depression after imaging visit or censoring at the end of follow-up or death. The Cox model was adjusted for the same covariates included in the primary analytical models. Proportional hazards assumptions were assessed using Schoenfeld residuals and global tests.

## Findings

### Sample characteristics

The primary cohort consisted of 1862 participants who met criteria for RD (table 1, online supplemental figure 1). The mean time interval between record of depressive episode and MRI was 7.76 years (SD 7.57 years). There were incomplete cognitive data for 696 participants from the RD cohort and 733 from the control cohort. After matching, participants with incomplete (n=1429/3724) as opposed to complete (n=2295/3724) cognitive data were not systematically different in terms of age or sex across groups (age: age*completion*group (F(1,3770)=0.09, p=0.77); sex: χ^2^=4.53, p=0.21); antidepressant use at imaging was also similar (35.8% in completers vs 33.2% in non-completers, see online supplemental table 1a). For potentially confounding variables and follow-up data, participants with RD with incomplete as opposed to complete data comprised a higher proportion of males and people endorsing white ethnicity, and more participants had post-university level education (see online supplemental table 1b).

**Table 1:** Characteristics and health factors of included participants

Characteristic	Matched control cohort (n=1862; mean (SD) unless stated)	Primary RD cohort (n=1862; mean (SD) unless stated)
Age (years)	52.7 (7.13)	52.7 (7.13)
Sex (%): Female; Male	1107 (59.4); 755 (40.6)	1107 (59.4); 755 (40.6)
Townsend Deprivation Index (%)		
1 (=least deprived)	866 (42.3)	805 (38.4)
2	680 (32.5)	725 (34.6)
3	240 (11.5)	247 (11.8)
4	51 (2.44)	78 (3.72)
5 (=most deprived)	5 (0.24)	7 (0.33)
Postsecondary education (%)		
Post university	875 (41.8)	873 (41.7)
A levels	271 (12.9)	249 (11.9)
O levels	347 (16.6)	385 (18.4)
CSEs	104 (4.97)	88 (4.20)
NVQ/HND	88 (4.20)	89 (4.25)
Other professional	80 (3.82)	91 (4.35)
None of the above	97 (4.63)	87 (4.15)
Ethnicity (%)		
White	1817 (86.8)	1836 (87.7)
Other ethnicity groups	45 (13.2)	26 (12.3)
Health factors		
Never smoker	1189 (63.9)	1050 (56.4)
Alcohol intake (mean and median, units/week (SD))	16.6; 12.6 (14.6)	16.0; 11.8 (15.2)
Body mass index (BMI) (mean, kg/m^2^ (SD))	26.0 (4.09)	27.3 (4.68)
Existing conditions		
Epilepsy	8 (0.43)	18 (1.00)
Cerebrovascular disease	26 (1.40)	45 (2.42)
Ischaemic heart disease	65 (3.49)	140 (7.52)
Previous cancer diagnosis	203 (10.9)	276 (14.8)
Depression factors		
Antidepressant use at imaging (%)	Not applicable	649 (34.9)

Results for other ethnicity groups conflated here consistent with UK Biobank small numbers reporting standardised operating procedure

CSE, Certificate of Secondary Education; HND, Higher National Diploma; NVQ, National Vocational Qualification; RD, remitted depression.

Compared with control participants, the RD group (n=1862) had a significantly higher average BMI, were more likely to be white, in less affluent socioeconomic groups, and to have pre-existing ischaemic heart and cerebrovascular disease and a previous diagnosis of cancer at the imaging visit (see table 1 and online supplemental table 2). One-third of the RD cohort also reported use of antidepressants at this point (34.9%). The RD cohort performed worse on cognitive tasks compared with controls when examined as a composite measure across all domains (F(1,2277)=6.7, p=0.01: composite z-score mean (SD): controls=0.159 (0.924); RD=0.065 (0.976)). At the individual domain level, there was a similar pattern with the largest difference in processing speed (mean z scores) (see online supplemental table 3). Lifetime number of depressed episodes for the RD cohort are detailed in online supplemental material (see online supplemental table 4a; data missing for 43% of RD cohort). As expected, an F41 diagnosis of anxiety disorder was more common in the RD cohort prior to imaging (33% RD; 3% controls) (see online supplemental table 4b).

Following imaging, 240 (13%) controls developed a first episode of depression, while 623 (33%) of the primary RD cohort experienced a relapse of depression (see online supplemental table 4C). Within the RD cohort, 231 individuals developing depression over the follow-up period were receiving antidepressants at the imaging visit (37% of RD cohort who relapsed; 12% of total RD cohort). In the adjusted Cox proportional hazards model accounting for temporal differences in longitudinal assessment, participants in the RD group were three times more likely than controls to develop depression during the follow-up period (HR=3.16 (95% CI 2.71 to 3.67), global proportional hazard assumption p=0.07; n=3724) (see figure 1B, online supplemental figure 2a and online supplemental table 5). Global model statistics indicated robust overall significance (likelihood ratio=275 on 12 df; Wald=245 on 12 df; log-rank=272 on 12 df; all p<2×10⁻¹⁶). Online supplemental figure 2b highlights that groups do not separate immediately.

### Cognitive markers of depression onset during follow-up

**Figure 2:**
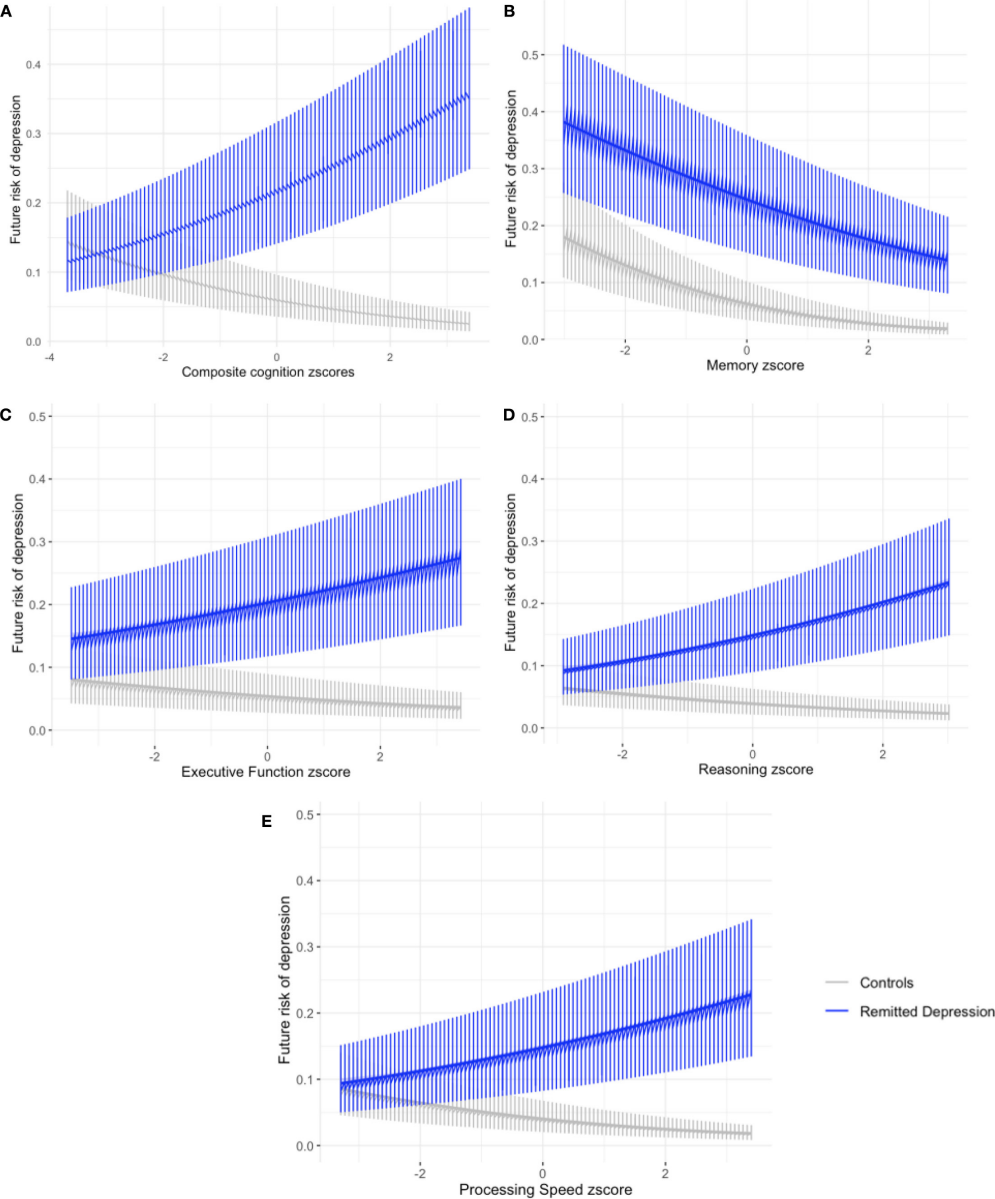
Future depression expressed as risk ≤1. Cognitive scores expressed as a z score. CIs around the mean risk shown. (**A**) Composite score across all dimensions; (**B**) memory scores (based on paired associate learning scores); (C) executive functioning scores (based on digit span, TMT B-A, tower arranging scores); (D) reasoning scores (based on fluid intelligence, matrix pattern puzzles scores) and (E) processing speed scores (based on reaction time, symbol digit substitution scores). Data included without imputation.

History of depression, risk of future depression and cognitive score at the imaging visit were significantly related (main effect of group: coefficient=−0.26, SE=0.11, z=−2.29, p=0.022; composite cognition score*group: coefficient=0.47, SE=0.14, z=3.4, p<0.001 (see figure 2A, online supplemental table 6)). For control participants, the risk of depression during the follow-up period was low overall, but higher for those with a relatively lower baseline composite cognitive score (mean (SD) depression risk as probability=0.13 (0.34), see figure 2; mean depression risk at mean±SD cognition z-score as estimated marginal means (EMM)=0.0020 (0.20%); −SD=0.0025 (0.25%), +SD=0.0015 (0.15%)). When analysing cognitive domains separately, lower performance on executive functioning, reasoning and processing speed were all associated with higher depression risk in controls (see figure 2C–E, online supplemental table 7b–d)). Paradoxically, the opposite pattern of associations was observed in participants with RD—that is, higher composite cognitive performance was associated with greater future depression risk (mean (SD) depression risk as probability=0.33 (0.47), see figure 2; mean depression risk at mean±SD cognition z-score as EMM=0.009 (0.9%); −SD=0.0074 (0.74%), +SD=0.011 (1.1%)). As seen with controls, scores for executive functioning, reasoning and processing speed all contributed to this finding.

Models without the inclusion of a group factor found no association between composite cognition score and future depression across the two groups (composite cognitive score (coefficient=0.03, SE=0.07, z=0.371, p=0.71), see online supplemental table 6 and online supplemental figure 3; for per domain see online supplemental table 7a–d).

Significant task measure by group interactions for correct digit symbol matches*group (p=0.002) and matrix pattern puzzles solved correctly*group (p=0.02) appeared to drive the differences seen at the summed dimension and overall composite score level (see online supplemental table 8 and online supplemental figure 4).

### Sensitivity analyses

Results for main analyses were similar when imputation was used to account for missing cognitive data (ie, composite cognitive scores: p=0.05; composite cognitive scores*group: p=0.003, see online supplemental table 6). Other sensitivity analyses of the primary outcome (association between cognitive performance and future depression) were consistent with the findings from the main analyses (see online supplemental table 9 and online supplemental figures 5 and 6). There were no significant interactions between any of the composite cognition score, group and TDI when this was included as an interaction term in the main model (composite cognition score*TDI: coefficient=0.057, SE=0.14, z=0.40, p=0.68; group*TDI: coefficient=−0.083, SE=0.17, z=−0.50, p=0.61; composite cognition score*group*TDI: coefficient=−0.070, SE=0.17, z=−0.41, p=0.68) (see online supplemental figure 6a,b). Logistic regression analysis assessing the interaction between ‘time between recorded depression and imaging’ and composite cognition in terms of the outcome of depressive relapse for the participants with RD suggested that the timing between the imaging visit and prior depression and composite cognition were not strongly associated with depressive relapse (composite cognition score*time between recorded depression and imaging: coefficient<−0.001, SE<0.001, z=−0.50993, p=0.62).

A slightly higher proportion of depressive relapse episodes occurred in the younger subset (64 years and under) of the study population, but there was no significant difference in odds of depression in control and RD groups comparing participants under 65 with those 65 and over (p=0.78; see online supplemental material, pp. 37–39). Regression analyses using these divided cohorts were limited by reduced power, but results were similar to main analyses, with the composite cognition z-score*group interaction significant in both subgroups (p<0.05, see online supplemental table 9). Precision (ie, tightness of CIs) appeared to be greater in the older subset despite the smaller number of participants included (see online supplemental figure 5). Findings were similar when we repeated main analyses with control participants defined by less stringent criteria (not in FO+HES cohort and no evidence of depression at imaging using PHQ-2) (composite cognition score*group: coefficient=0.494, SE=0.14, z=3.56, p=0.0003; pool for control participants 36 073 (less stringent) vs 34 252 (as used in main analyses)) (see online supplemental figure 1). 97% of 1862 control participants included in main analyses also met criteria for this ‘less stringent’ condition.

As post hoc robustness analyses, we evaluated (1) the effect of antidepressant use on cognitive scores at the imaging visit, (2) the proportions of participants who overlapped between different RD cohorts depending on definition (FO+HES/GP data/Probable Depression algorithm) and (3) the demographics between RD and control participants who had a future episode of depression. Antidepressants at baseline and imaging did not appear to have a significant effect on summed cognitive scores at imaging (p>0.2; see online supplemental table 10). Proportions of participants who overlapped between primary and other cohorts were similar (see online supplemental table 11). Demographics were similar between control and participants with RD who experienced an episode of depression after the imaging visit (see online supplemental table 12).

Differences between GP/Probable Depression cohorts and the FO+HES primary cohort compared with their respective matched controls are outlined in online supplemental table 13a–d). The general pattern of results was similar across both secondary cohorts (ie, GP records; Probable Depression) compared with the primary cohort (FO+HES) for the primary outcome (association between cognitive performance and future depression). However, results indicated that these secondary cohorts were lower power and less precise with broader CIs than seen in primary analyses when compared with their matched controls cohorts in terms of future risk of depression modelled using both composite cognitive scores (online supplemental figure 7) and individual cognitive dimensions (online supplemental figure 8).

### MRI markers and associations with cognition and future depression

Of the 1862 FO+HES RD cohort used for main analyses, 1554 participants had cognitive data as well as full data for MRI and control variables (see online supplemental table 1b) for participant characteristics of this cohort subset). This subset was used for secondary analyses involving MRI data after matching 1:1 to control participants using the same procedure as for primary analyses.

Compared with the control group, the RD group had greater head movement and reduced mean grey matter volume. Other imaging parameters were not significantly different between groups (see online supplemental table 14). There were no significant differences in grey matter volumes of structural regions of triple network (pre-specified) between participants with RD when compared at the imaging assessment (see online supplemental table 15a and b)).

**Figure 3:**
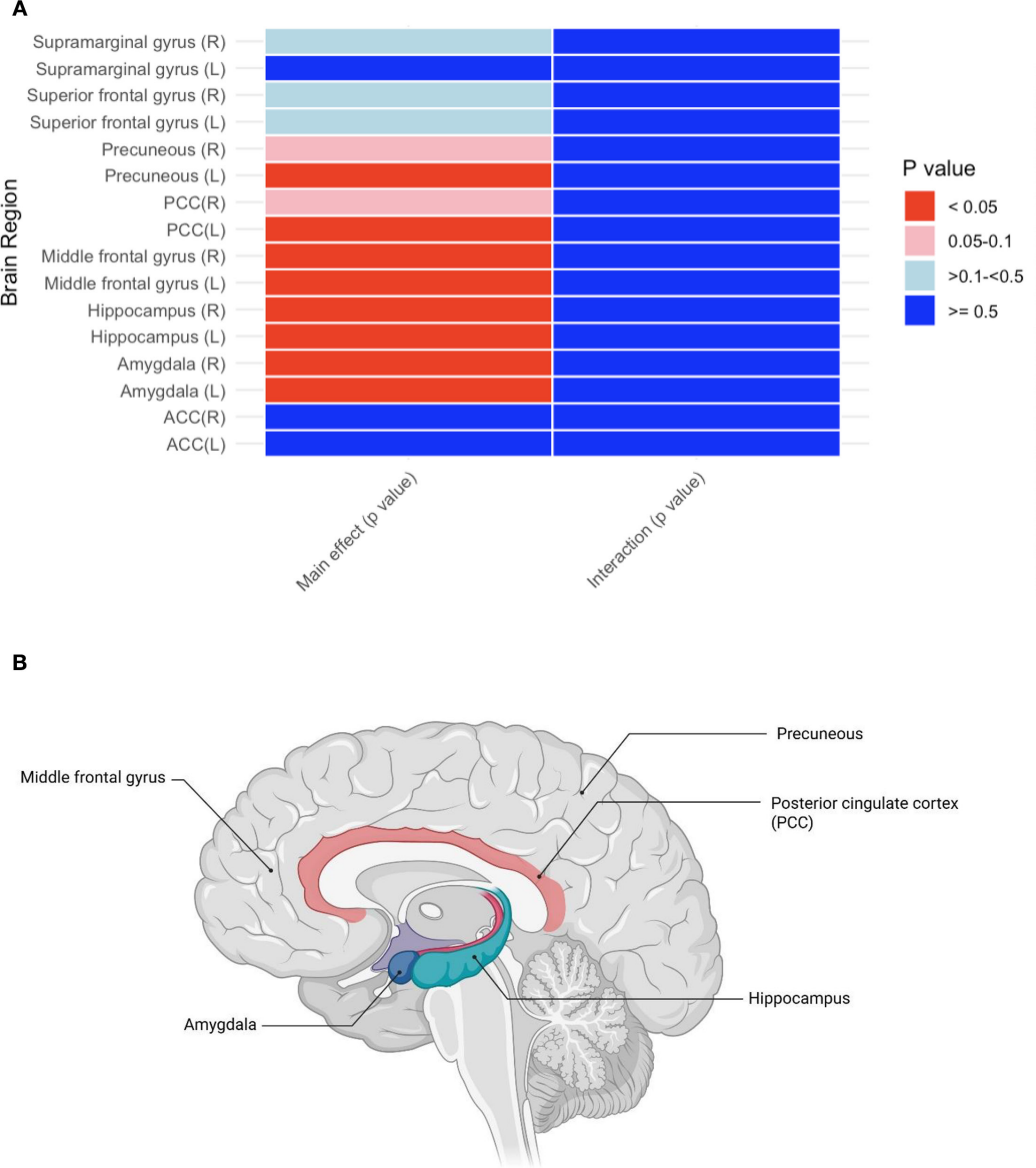
(**A**) P value where p<0.05 is significant, shown corrected for FDR for main effect of region and for region*group interaction. Red tone colour indicates p<0.05 as shown by colour bar. Data included without imputation. (**B**) Created in Biorender.com. FDR, false discovery rate; ACC, anterior cingulate cortex.

Increased volume of the amygdala, hippocampus and middle frontal gyrus bilaterally and the left precuneus and left posterior cingulate cortex (PCC) were associated cross-sectionally with better composite cognitive performance across all participants (see figure 3 and online supplemental table 16). These regions are considered part of the default mode network.

Structural measures of the triple network were not associated with depression risk among control participants or individuals with RD (see online supplemental table 17). Similar findings were observed for functional connectivity measures of the triple network (see online supplemental tables 18 and 19).

## Discussion

As hypothesised, lower cognitive performance predicted a higher risk of future depression in participants without a prior history. Those with past depression (RD) showed poorer cognitive function and reduced grey matter volume on average compared with matched controls and were three times more likely than control participants to experience a depressive episode during follow-up. Surprisingly, those individuals with RD with the highest cognitive performance appeared at greatest risk of relapse. MRI measures did not explain this vulnerability.

People who have experienced depression have previously been shown to be at higher risk of further episodes compared with controls.^23^ We replicated this finding in our results: 33% of participants with RD had a subsequent episode in follow-up compared with 13% of matched controls; and survival analysis confirmed this threefold risk accounting for differing periods of risk. A goal of individualised risk prediction is to identify those within this higher risk RD cohort that are most at risk to enable targeted intervention and potentially improved mental health and functional outcomes. Cognitive functioning has been suggested as a possible risk marker of those at highest risk for relapse in RD^2^: there is consistent evidence that at least 40% of people with RD perform poorly in terms of cognition across a variety of behavioural tasks,^4 5^ with these impairments worsening with subsequent depressive episodes.^4 5 10^ In this study, we identified that cognitive performance appears to be associated with risk of future depression differently in those with previous depression compared with matched never-depressed controls. That is, lower baseline composite cognitive performance was associated with higher risk of future depression in people who had not experienced depression before, but that relationship was reversed for those with RD, that is, higher cognitive performance across a range of tasks was associated with higher future depressive risk. Furthermore, understanding which cognitive domains may be of relevance for longitudinal risk in RD has been relatively underexplored. In our study, consistent with meta-analyses involving participants,^24^ we found that cognitive performance on the memory task showed limited (non-significant) impact on future depressive risk between the groups compared with other cognitive dimensions (executive function, processing speed, reaction time).

This study is a novel first step to triangulate cognitive performance and MRI findings with longitudinal depression risk in RD. Our primary analysis findings (ie, in RD, higher composite cognitive scores were associated with an increased risk of a subsequent depressive episode) were counter to our hypothesis and might seem counterintuitive. This relationship was not evident in control participants, suggesting it was likely not related to the UKB cohort per se. However, it is interesting to consider why a difference may exist between participants with RD and controls in terms of the relationship between baseline cognitive functioning and future risk of depression. One possible explanation is that adults with RD (who are at greater risk of depression than controls) and with a higher level of cognitive functioning may be more likely than participants with RD at a lower cognitive level to notice and report future lower mood symptoms. That is, impaired cognition may increase risk up to a threshold, beyond which people are too impaired to reflect and report symptoms (see online supplemental table 3) for the sample cognitive performance distribution). Alternatively, it is possible that control participants who went on to develop depression during the follow-up period may have been experiencing subclinical depressive symptoms at the imaging visit that impacted on their cognitive performance. However, a sensitivity analysis using only control participants scoring below threshold on both PHQ-2 questions (the best measure of depressive symptomatology available at the imaging visit) did not suggest different findings. Furthermore, although we excluded participants with pre-existing cardiovascular or neurological disorders or those who developed dementia during the follow-up period, this ‘late onset depression’ (new depression during follow-up in our participants relates to later life as UKB participants are older than 40 on initial assessment) may be more aligned with underlying organic causes and thus be more closely associated with worse cognitive performance. Socioeconomic factors are known to be linked with depression and associated with a poorer prognosis independent of the type of treatment received.^25^ However, within the older UKB sample, Cullen and colleagues have found previously that cognitive performance in those at greater risk of depression (due to familial risk) was not influenced by educational or socioeconomic factors.^22^ This is consistent with our sensitivity analysis finding limited evidence for the impact of socioeconomic status on our findings or differences in socioeconomic status across our groups. A future study assessing cognitive functioning longitudinally alongside evolving risk of future depression may be able to provide more clarity for some of these hypotheses. Other psychosocial risk markers (such as ongoing stress or social support) and subthreshold depressive symptoms may be important in the context of cognitive performance in future depression but the data capture of this was limited at the first imaging visit within the UKB.

The relationship between future relapse risk in RD, abnormal functioning of the triple network, and cognitive performance within RD has been unclear, although both impairments in cognition and the triple network (DMN, cEN, SN) have been suggested as vulnerability markers for future episodes.^16 17^ In RD, functional changes within and between neural networks including the triple network^13 14 26^ have been found to exist, and in healthy volunteers, connections involving these specific networks also directly link to cognitive performance.^15^ We identified that increased volume of regions of the DMN (amygdala, hippocampus, middle frontal gyrus, precuneus and PCC) were associated with improved cross-sectional cognitive performance across all participants, but this was not specifically associated with a prior history of depression (after controlling for baseline MRI measures). We also demonstrated that compared with the control group, the RD group had reduced grey matter overall. Taken together, this may explain why our previously depressed cohort performed significantly worse on cognitive tasks compared with controls matched for age and sex.

Our analyses had several strengths. The primary RD cohort, using FO+HES, was carefully constructed with participants that had met ICD-10 diagnostic criteria for depression. We conducted multiple sensitivity, robustness and subgroup analyses to confirm and explore our findings, and imputation to account for any missing cognitive data across participants when calculating the composite cognition score. Sensitivity and robustness analyses indicate that our results are not explained by other factors, such as the presence of cerebrovascular disease, a close family history of depression, age or the use of antidepressants. Checks completed prior to imputation confirmed that missing cognitive data were missing at random, and analyses with and without imputation were similar, providing reassurance that missing data had minimal impact on results. Consistent with this, potential reasons for incomplete cognitive data include, but are not limited to, assessment logistics, such as technical issues, and participant burden concerns or appointment time constraints.

When assessing the risk of depression over the period of follow-up across groups, it is important to recognise that for each individual the recorded date of depression using the MHFU or Online follow-up questionnaire or HES data is an estimated date of illness. When RD was defined using other UKB methodologies (using GP records or using the UKB ‘Probable Depression’ algorithm), results were similar but less precise, supporting findings and suggesting that larger sample sizes are likely to be required if these less stringent definitions are used compared with those based on defining RD using FO+HES. Interpretation of these findings should consider that the RD group may include individuals with concurrent or overlapping anxiety features, reflecting the substantial symptom and diagnostic overlap between these conditions in routine clinical data. rsfMRI findings may have been affected by limited power due to only a subgroup of participants having full MRI data. Future analyses should also consider including the number of depressive episodes into results and longitudinal assessment of cognition alongside evolving depression risk. As a resource, the UKB has general limitations in its representativeness of participants, particularly in terms of general health, ethnicity and socioeconomic status compared with the general population.^27^ In particular, we note that over 95% of our study population were white, potentially limiting generalisability of our findings. Mean number of units per week for both the primary RD and matched control groups is greater than recommended by UK health professionals, although there was significant variability in the data and median values were within recommended guidelines. Alcohol use can affect cognitive performance and depression and thus we accounted for alcohol use within our statistical models. Our findings were similar across both <65 and 65+ participants, but precision appeared to be greater for the 65+ subset. Although we should be cautious of post hoc analyses in smaller sub-samples, it indicates that age may be relevant when considering cognitive scores and future risk of depression outside of neuropsychiatric illness in terms of future research.

Using a large national cohort, we confirmed RD is a high-risk group for future depression. Age-matched and sex-matched control participants were at greatest risk of subsequent depression if baseline cognitive performance was poorer, but this relationship was reversed in RD. Ongoing exploration is required to explore methods of targeted intervention based on the specific cognitive profile of RD to support those with previous episodes of depression to stay well.

## Clinical implications

Personalised risk factor assessment for depression is required. Our findings suggest this may be dependent on depression history. Those with no previous history of depression are at higher risk of future depression when cognitive performance is lower at baseline. RD is a high-risk group for future depression, and those with relatively higher cognitive performance may be more likely to report future depressive symptoms.

## Supplementary material

10.1136/bmjment-2025-302332 online supplemental file 1

## Data Availability

Data may be obtained from a third party and are not publicly available.
