# Supplementary material for: Cognition and future depression: associations with risk in those with and without a history of depression
Source: BMJ Ment Health. 2026 May 7;29(1):e302332. doi: 10.1136/bmjment-2025-302332 (PMC13150916; doi:10.1136/bmjment-2025-302332)
Supplement: online supplemental file 1 [file bmjment-29-1-s001.pdf]

Supplementary Material

Contents

Supplementary Material 1
Supplementary Methods 4
MRI methods 4
Overview of cohort inclusion criteria 5
Overview of depression assessment during the follow up period (all cohorts) 5
Codes used for definition of cohorts and antidepressant use within the UK Biobank 6
Analytic models relevant for each research question as pre-specified 9
Potential confounds (controlled for in regression analyses) 11
Health exclusions (participants excluded if occur at or within 2 years after imaging) 11
Prior checks to confirm validity of imputation 12
Supplementary Results 22
Supplementary Figure 1: Flow diagram 22
Supplementary Table 1(a): Comparison between those with complete and incomplete cognitive data in the FO+HES RD / control cohorts for age and sex 23
Supplementary Table 1(b): Characteristics at baseline and health factors at imaging of participants with incomplete data for control variables and follow-up time 24
Supplementary Table 1(c): Characteristics at baseline and health factors at imaging of participants with complete MRI data 26
Supplementary Table 2: Comparison between remitted depressed cohort with complete data for control variables (n=1862) and matched control cohort (n=1862) for FO+HES cohort 28
Supplementary Table 3: Behavioural cognitive functioning at imaging (without imputation) 29
Supplementary Table 4(a): Lifetime of depressed periods (self-report; f.20442 from the 2016-2017 online follow-up) for the FO+HES remitted depressed (RD) cohort 30
Supplementary Table 4(b): Anxiety Disorder (First Occurrences ICD-110 F41) for the FO+HES remitted depressed (RD) and control cohorts 30
Supplementary Table 4(c): Sources of longitudinal data for future depressive episodes (n; %) 30
Supplementary Figure 2(a): Adjusted hazard ratios from proportional hazards cox model for Figure 1(B) 31
Supplementary Figure 2(b): Kaplan-Meier survival curve as Figure 1(B) (truncated to 2 years follow up) 31
Supplementary Table 5: Proportional hazards for Figure 1(B) 32
Supplementary Table 6: Risk of future depressive episodes and impact of composite cognitive scores at imaging visit 33

Supplementary Figure 3: Future depression risk relating to cognition scores at imaging visit for all comers (model without group) 33
Supplementary Table 7(a): Risk of future depressive episodes and impact of memory score (n=3724; RD:controls=1862:1862) 34
Supplementary Table 7(b): Risk of future depressive episodes and impact of executive function (EF) score (n=2310; RD:controls=1174:1136) 34
Supplementary Table 7(c): Risk of future depressive episodes and impact of reasoning score (n=2617; RD:controls=1315:1302) 34
Supplementary Table 7(d): Risk of future depressive episodes and impact of processing speed (PS) score (n=2639; RD:controls=1326:1313) 34
Supplementary Table 8: Risk of future depressive episodes and impact of individual cognitive tasks 35
Supplementary Figure 4: Future depression risk relating to cognition scores at imaging visit for individual tasks 36
Results from Sensitivity Analyses for Primary analyses (using FO+HES cohort) 37
Supplementary Table 9: Sensitivity analyses for primary outcome 38
Supplementary Figure 5: Future depression risk relating to composite cognition scores at imaging in the primary cohort: (A) 64 and under subset; (B) 65 and over subset 39
Supplementary Figure 6(a): Future depression risk relating to composite cognition scores at imaging in the primary cohort when Townsend Deprivation Index (TDI) included as an interaction term in the model 40
Supplementary Figure 6(b): Mean composite cognition by Townsend Deprivation Index (TDI) quintile and group (see Table 1 for n) 40
Results from Sensitivity Analyses for Primary outcome: characteristics for GP cohort and Probable Depression cohort 41
Results from Sensitivity Analyses for Primary outcome: (i) Summed cognitive score impact on future risk of depression for GP cohort: 42
Results from Sensitivity Analyses for Primary outcome: (ii) Summed cognitive score results for Probable Depression cohort: 42
Supplementary Figure 7: Future depression risk relating to composite cognition scores at imaging: (A) using GP records to define past depression; (B) using the Probable Depression algorithm to define past depression 43
Supplementary Figure 8: Dimensional comparison across the primary and secondary cohorts 44
Robustness check 1) Effect on antidepressant use on cognition at baseline / imaging within remitted depression group 48
Supplementary Table 10: Proportion of remitted depression cohort using antidepressants 48
Robustness check 2) Overlap between cohorts 48
Supplementary Table 11: Overlap between primary and secondary cohorts 48
Robustness check 3) Relapse rate across different confounders 48
Supplementary Table 12: Demographics of participants in primary cohort who developed depression after imaging (RD cohort = 623; control cohort = 240) 49

Supplementary Table 13(a): Comparison between remitted depressed cohort with complete data for control variables (n=1039) and matched control cohort (n=1039) for GP cohort 50
Supplementary Table 13(b): Comparison between remitted depressed cohort with complete data for control variables (n=2252) and matched control cohort (n=2252) for Probable Depression cohort 50
Supplementary Table 13(c): Differences between RD and matched control primary cohorts when compared with other secondary RD and matched control cohorts 51
Supplementary Table 13(d): Differences with other cohorts in terms of cognitive functioning at the imaging visit 52
Supplementary Table 14: Imaging parameters at baseline (n=1554) 53
Supplementary Table 15(a): Regional structural volumes of the Triple Network for RD cohort with full imaging data (Group 1; n=1554) and matched control cohort (Group 0; n=1554) 54
Supplementary Table 15(b): Group differences in structural imaging for RD cohort with full imaging data (n=1554) and matched control cohort (n=1554) 55
Supplementary Table 16: Composite cognition scores and association with cross-sectional brain regional volumes across all participants (n=1554) 56
Supplementary Table 17: Impact of brain regional volumes within Triple Network on future risk of depression for RD (n=1554) and matched control cohorts (n=1554) 57
Supplementary Table 18(a): Impact of rsfMRI functional connectivity (nodes) on cognitive scores within Triple Network 58
Supplementary Table 18(b): Impact of rsfMRI functional connectivity (edges) on cognitive scores within Triple Network 59
Supplementary Table 19(a): Impact of rsfMRI functional connectivity (nodes) on future risk of depression 62
Supplementary Table 19(b): Impact of rsfMRI functional connectivity (edges) on future risk of depression 63
STROBE Statement—checklist of items that should be included in reports of observational studies 66

Supplementary Methods

MRI methods

The UK Biobank team generated the brain imaging data which was accessible to researchers. The exclusion criteria established by the UK Biobank team for MRI scanning. include standard MRI safety/quality criteria, such as exclusions for metal implants, recent surgery or health conditions problematic for MRI scanning, such as claustrophobia. Additionally, raw MRI data that had incorrect dimensions, or were corrupted, missing, or otherwise unusable were not processed and available for inclusion.

The brain imaging data were preprocessed by a fully automated processing pipeline primarily centered around FSL software [1]. The imaging modalities within the UK Biobank comprised T1-weighted, T2-weighted flair, diffusion MRI (dMRI), susceptibility-weighted imaging (SWI), task functional MRI timeseries data (tfMRI), and resting-state functional MRI timeseries data (rsfMRI) [2]. T1-weighted MPRAGE and T2-weighted FLAIR volumes were obtained at 1×1×1mm (208×256×256 field of view [FOV] matrix) and 1.05×1×1mm (192×256×256 FOV matrix) respectively. Linear and non-linear registration to MNI152 "nonlinear 6th generation" standard-space was conducted using FMRIB's Linear Registration Tool (FLIRT) and FMRIB's Nonlinear Image Registration Tool (FNIRT) respectively. Additionally, brain extraction was performed using the Brain Extraction Tool (BET), followed by defacing and segmentation into tissue types using FMRIB's Automated Segmentation Tool (FAST). Grey matter was extracted from FAST and the estimation of the grey matter was performed for a total of 139 regions of interest (ROIs) defined by the Harvard-Oxford cortical and subcortical atlases and the Diedrichsen cerebellar atlas. Subcortical volumes were generated by using FMRIB's Integrated Registration and Segmentation Tool (FIRST) and estimation was done using the population priors on shape and intensity variation across subjects. Image segmentation was performed to identify white matter hyperintensities (WMH). Additionally, periventricular WMH (pWMH) and deep WMH (dWMH), were defined based on subsets of total WM hyperintensities.

Resting state functional MRI (rsfMRI) were also obtained at 2.4×2.4×2.4mm (88×88×64 FOV matrix). On a preprocessed sample of 4162 participants, grouped average independent component analysis (ICA) was carried out using MELODIC [3, 4]. ICA was performed with dimensionality set to 25 and 100, which resulted in 21 and 55 components, respectively, after discarding the noise components. These 21×21 and 55×55 partial correlation matrices were used as measurements of functional connections. The ICA maps were mapped on each participant's rsfMRI timeseries data in order to acquire one representative node timeseries per ICA component for each subject. These network 'nodes' are a measure of within-network functional connectivity. Subject-specific network-matrices ('edges') were also extracted from the node timeseries that provide a measure of functional connectivity between the nodes [5]. Previous UK Biobank studies [15, 21] suggested networks of interest (NOI) for cognition in depression include the following 11 components (nodes) based on "triple network" of default mode network (DMN), central executive network (cEN / FPN) and salience network (SN): (i) DMN = 1, 7, 9, 14, 20; (ii) cEN / FPN = 5, 6, 16, 21 (5, 6 and 21 are part DMN, part cEN); (iii) SN = 3, 13 (13 is part DMN, part SN). 54 edges involving these 11 nodes were taken from https://www.fmrib.ox.ac.uk/datasets/ukbiobank/group_means/edge_list_d25.txt). Structural regions of interest relating to the "triple network" as below were pre-specified as amygdala, anterior cingulate cortex, hippocampus, medial frontal gyrus, precuneous, posterior cingulate cortex, supramarginal gyrus, superior frontal gyrus (bilaterally).

MRI methods references

1. Jenkinson, M., et al. (2012). FSL. NeuroImage, 62(2), 782–790. https://doi.org/10.1016/j.neuroimage.2011.09.015

2. Miller, K. L, et al. (2016). Multimodal population brain imaging in the UK Biobank prospective epidemiological study. Nature neuroscience, 19(11), 1523–1536. https://doi.org/10.1038/nn.4393

3. Alfaro-Almagro, F. et al. (2018). Image processing and quality control for the first 10,000 brain imaging datasets from UK Biobank. Neuroimage 166, 400–424

4. Beckmann, C. F., & Smith, S. M. (2004). Probabilistic independent component analysis for functional magnetic resonance imaging. IEEE transactions on medical imaging, 23(2), 137–152. https://doi.org/10.1109/TMI.2003.822821

5. Smith, S. M., et al. (2013). Functional connectomics from resting-state fMRI. Trends in Cognitive Sciences, 17(12), 666–682. https://doi.org/10.1016/j.tics.2013.09.016

Overview of cohort inclusion criteria

FO+HES (First Occurrences + Hospital Episode Statistics) remitted depression cohort: ICD depressive episode of moderate / severe severity occurring up to 15 years before the first imaging visit AND scoring 1 (not at all) or 2 (several days but less than half the days) on each of the two self-report Patient Health Questionnaire (PHQ)-2 core questions (f.2050.2.0; f.2060.2.0).

General Practice (GP) remitted depression cohort: Depression codes for moderate to severe ICD-10 depression for those with linked primary care records AND scoring 1 (not at all) or 2 (several days but less than half the days) on each of the two self-report Patient Health Questionnaire (PHQ)-2 core questions as above.

The “Probable depression” remitted depression cohort: “Probable depression” using this UKB-derived algorithm derived from self-report variables AND scoring 1 (not at all) or 2 (several days but less than half the days) on each of the two self-report Patient Health Questionnaire (PHQ)-2 core questions as above.

Control cohort: Matched 1:1 to sex and age +/- 2 years for each participant from each cohort above using a pool of participants with required data AND not included in any of three RD cohorts AND no antidepressant use at the imaging visit (using tabular antidepressant codes).

Overview of depression assessment during the follow up period (all cohorts)

(i) presence of a HES depression code (data available until April 2023) after the imaging time point (f.53.2.0); OR

(ii) a PHQ-9 score greater than 9 (f.120104.0.0 to f.120112.0.0 inclusive; collected from 2019-2023) if occurred after imaging visit (f.120128.0.0 > f.53.2.0); OR

(iii) age at last episode of depression on Mental Health Follow Up (f.20434.0.0; collected 2016-2017) if greater than age at imaging visit; OR

(iv) recent depression recorded at the Online Follow Up visit (f.120044.0.0; collected from 2019-2023) if occurred after imaging visit (f.120128.0.0 > f.53.2.0).

Codes used for definition of cohorts and antidepressant use within the UK Biobank

FO+HES codes

Code	Variable	Source
130894	First report of ICD-10 depressive disorder	First Occurrences
130896	First report of ICD-10 recurrent depressive disorder	First Occurrences
41270	HES_summary_diagnosis_ICD-10	Hospital Episode Statistics

GP codes

Code	Variable
1465	H/O: depression
62T1.	Postnatal depressive disorder
E112.	Depression: [single major episode] or [agitated] or [endogenous (including first episode)]
E1120	Single major depressive episode, unspecified
E1122	Single major depressive episode, moderate
E1123	Single major depressive episode, severe, without mention of psychosis
E1124	Single major depressive episode, severe, with psychosis
E1125	Single major depressive episode, in partial or unspecified remission
E1126	Single major depressive episode, in full remission
E112z	Single major depressive episode NOS
E113.	Recurrent depression: [major episode] or [endogenous]
E1130	Recurrent major depressive episodes, unspecified
E1132	Recurrent major depressive episodes, moderate
E1133	Recurrent major depressive episodes, severe, without mention of psychosis
E1134	Recurrent major depressive episodes, severe, with psychosis
E1135	Recurrent major depressive episodes, in partial or unspecified remission
E1136	Recurrent major depressive episodes, in full remission
E1137	Recurrent depression
E113z	Recurrent major depressive episode NOS
E11z2	See X00SU
E130.	Reactive depressive psychoses
E135.	See X00SQ
E2003	See X00Sb
E204.	(Neurotic depression reactive type) or (postnatal depression)
.E222	See X00SR
E2B..	Depressive disorder NEC
E2B0.	Postviral depression
E2B1.	Chronic depression
Eu32.	See XE1Y0

Eu321	[X]Moderate depressive episode
Eu322	[X] Severe depressive episode without psychotic symptoms: (& [single episode agitated depression] or [single episode major depression] or [single episode vital depression])
Eu323	[X] Severe depressive episode with psychotic symptoms: (& single episode of [major depression] or [psychogenic depressive psychosis] or [psychotic depression] or [reactive depressive psychosis])
Eu32y	[X] Depression: [other episodes] or [atypical] or [single episode masked NOS]
Eu32z	[X] (Depression: [episode, unspecified] or [NOS (& reactive)] or [depressive disorder NOS]
Eu325	See XSGok
Eu326	See XSGol
Eu327	See XSGom
Eu328	See XSGon
Eu33.	[X]Recurrent depressive disorder (& [episodes of depressive reaction] or [episodes of psychogenic depression] or [episodes of reactive depression] or [seasonal depressive disorder])
Eu331	[X]Recurrent depressive disorder, current episode moderate
Eu332	[X]Depression without psychotic symptoms: [recurrent: [major] or [manic-depressive psychosis, depressed type] or [vital] or [current severe episode]] or [endogenous]
Eu333	[X] Depression with psychotic symptoms: [recurrent: (current episode severe) or (manic-depressive, depressed type) or (severe major episodes) or (severe episodes)] or [endogenous]
Eu334	[X]Recurrent depressive disorder, currently in remission
Eu33y	[X]Other recurrent depressive disorders
Eu33z	[X] Depression recurrent: [unspecified] or [monopolar NOS]
.E35	See XE1aY
Eu412	[X]Mixed anxiety and depressive disorder
X00SO	Depressive disorder
X00SQ	Agitated depression
X00SR	Endogenous depression
X00SS	Endogenous depression first episode
X00SU	Masked depression
X00Sb	Mixed anxiety and depressive disorder
X40Dm	Severe postnatal depression
XE1Y0	Single major depressive episode
XE1Y1	Recurrent major depressive episodes
XE1YC	Reactive depression
XE1aY	Depression: [reactive (neurotic)] or [postnatal]
XM1GC	Endogenous depression - recurrent
XE1ZY	[X]Severe depressive episode without psychotic symptoms
XE1ZZ	[X]Severe depressive episode with psychotic symptoms
XE1Za	[X]Other depressive episodes
XE1Zb	[X]Depressive episode, unspecified
XE1Zc	[X]Recurrent depressive disorder

XE1Zd	[X]Recurrent depressive disorder, current episode severe without psychotic symptoms
XE1Ze	[X]Recurrent depressive disorder, current episode severe with psychotic symptoms
XE1Zf	[X]Recurrent depressive disorder, unspecified
XSGok	Major depression
XSGol	Moderate major depression
XSGom	Severe major depression without psychotic features
XSGon	Severe major depression with psychotic features

Probable depression (UKB derived algorithm) codes

Researcher-Derived Data Fields

Field Descriptions and Derivation for variables related to bipolar disorder, major depression status and neuroticism score

**UK Biobank Project ID**	7155	**From Publication**	Prevalence and Characteristics of Probable Major Depression and Bipolar Disorder within UK Biobank: Cross-Sectional Study of 172,751 Participants
**Principal Investigator**	Prof. Jill Pell		
**Institution**	Institute of Health & Wellbeing, University of Glasgow	**Citation**	Smith *et al.* (2013) PLOS ONE. 8: e75362
**Keywords**	Mental Health, Bipolar, Neuroticism, Depression	**DOI**	10.1371/journal.pone.0075362

Major Depression Status Fields (3)

20123 Derived field: Single episode of probable MD

20124 Derived field: Probable recurrent MD (moderate)

20125 Derived Field: Probable recurrent MD (severe)

Lifetime major depression (MD) status of participant defined from the touchscreen questionnaire at baseline

Possible Values [1]
For each 20123, 20124, 20125
1 = history of major depression

T/Screen

4598 Ever depressed for a whole week
4609 Duration of longest period of depression (weeks)
4620 Number of depressive episodes ≥ 1 week duration
4631 Ever unenthusiastic/disinterested for a whole week
5375 Duration of longest period of unenthusiasm/disinterest (weeks)
5386 Number of unenthusiastic/disinterested episodes ≥ 1 week duration
2090 Seen Doctor (GP) for nerves, anxiety, tension or depression
2100 Seen psychiatrist for nerves, anxiety, tension or depression

Availability
Subset of the cohort (n = 188,336)
Derived at baseline only

Each of the three depression states were defined based on a number of criteria:

Criteria
(1) Ever felt depressed for a whole week
(2) Ever disinterested or unenthusiastic for a whole week
(3) Only 1 episode
(4) ≥ 2 episodes
(5) Episode lasted ≥ 2 weeks
(6) Ever seen a GP for nerves, anxiety, tension or depression
(7) Ever seen a psychiatrist for nerves, anxiety, tension or depression

Definitions
Single Probable MD Episode:
{[(1) AND (3) AND (5) AND [(6) OR (7)]} OR {(1) AND (3) AND (5) AND [(6) OR (7)]}
Probable Recurrent MD (moderate):
[(1) OR (2)] AND (4) AND (5) AND (6)
Probable Recurrent MD (severe):
[(1) OR (2)] AND (4) AND (5) AND (7)

Codes used to ascertain antidepressant use within the UK Biobank (tabular data):

Variable name	Code	Generic name (if different)	Class of antidepressant
mirtazapine	1141152732		NASSA
zispin 30mg tablet	1141152736		NASSA
mianserin	1140879556		NASSA
zyban 150mg m/r tablet	1141176858	Bupropion	Antidepressant
tranylcypromine	1140867914		MAOi
parnate 10mg tablet	1140867916	Tranylcypromine	MAOi
moclobemide	1140867920		MAOi
manerix 150mg tablet	1140867922	Moclobemide	MAOi
phenelzine	1140910704		MAOi
reboxetine	1141151978		Noradrenaline reuptake inhibitor
edronax 4mg tablet	1141151982	Reboxetine	Noradrenaline reuptake inhibitor
trazodone	1140879634		Serotonin modulators

nefazodone	1140917460		Serotonin modulators
venlafaxine	1140916282		SNRI
nortriptyline	1140867818		SNRI
clomipramine	1140879620		SNRI
efexor 37.5mg tablet	1140916288	Venlafaxine	SNRI
citalopram	1140921600		SSRI
fluoxetine	1140879540		SSRI
sertraline	1140867878		SSRI
paroxetine	1140867888		SSRI
escitalopram	1141180212		SSRI
seroxat 20mg tablet	1140882236	Paroxetine	SSRI
prozac 20mg capsule	1140867876	Fluoxetine	SSRI
cipralex 5mg tablet	1141190158	escitalopram	SSRI
lustral 50mg tablet	1140867884	Sertraline	SSRI
fluvoxamine	1140879544		SSRI
felicium 20mg capsule	1141170866	Fluoxetine	SSRI
oxactin 20mg capsule	1141174756	Fluoxetine	SSRI
ranflutin 20mg capsule	1141185124	Fluoxetine	SSRI
amitriptyline	1140879616		TCA
imipramine	1140879630		TCA
dothiepin	1140879628		TCA
butriptyline	1140856074	Butriptyline	TCA
evadyne 25mg tablet	1140856076	Butriptyline	TCA
maprotiline	1140879552		TCA
desipramine	1140879624		TCA
protriptyline	1140879632		TCA
thaden 25mg capsule	1141171824	Dosulepin	TCA

Analytic models relevant for each research question as pre-specified

Primary analyses:

Are cognition and depressive relapse associated in remitted depression (RD), and how does this differ from never-depressed (ND) population

- Full sample: Depressive episode after imaging visit ~ Cognitive scores (*total score*)
- ND vs RD: Depressive episode after imaging visit ~ Group*Cognitive scores (*total score*) – PRIMARY OUTCOME

Secondary analyses:

Are there any significant differences between remitted depression and control cohorts for average grey matter volume in key regions of triple network, and do any differences have an impact on (i) concurrent cognitive scores and (ii) relapse

- ND vs RD: Cognitive scores (total score) ~ Group*structural differences
- ND vs RD: Depressive outcome after imaging visit ~ Group*structural differences

Are previous associations found between resting state functional connectivity (rsFC) of Triple Network and cognition in depressed volunteers reproduced in this RD population?

- ND vs RD: Cognitive scores (total score) ~ Group*rsFC (*for networks of interest – amplitudes (nodes) & edges*)

Is rsFC within the Triple Network and depressive relapse associated in RD, and how does this differ from ND population?:

- Full sample: Depressive episode after imaging visit ~ rsFC *(triple network nodes & edges)*
- ND vs RD: Depressive episode after imaging visit ~ Group*rsFC (*triple network nodes & edges*) – PRIMARY OUTCOME

Potential confounds (controlled for in regression analyses)

- Demographics (baseline where crystallised to capture all data or imaging visit tabular data)
    - sex (M/F) (31.0 TD) – part of matching process
    - age in years at time of assessment (21003.2 TD) – part of matching process
        - Pre-processed into age^2 and age^3, and age × sex, age^2 × sex for imaging analyses (in addition to matching for age and sex)
    - dates of assessment (53.0/2 TD)
    - assessment centre site (54.2 TD)
    - self-identified ethnic group: White, Black / Black British, Asian & Mixed & Other (21000.0 TD)
- SES / education (baseline where crystallised or imaging visit)
    - self-report of highest qualification (university degree / A levels / GCSEs / CSE / NVQ / professional quals / none) (6138.0 TD (array of 7))
    - neighbourhood deprivation level at recruitment (189)
        - Pre-processed into quintiles; 1 = least deprived and 5 = most deprived)
- Imaging – for imaging analyses *(imaging visit tabular data)*
    - mean rfmri head motion, averaged across space and time points (25741.2.0 TD)
    - structural MRI
        - total brain volume (normalised for head size, 25009.2.0 TD)
        - GM volume (normalised for head size, 25005.2.0 TD)
        - WM volume (normalised for head size, 25007.2.0 TD)
        - CSF volume (normalised for head size, 25003.2.0 TD)
- General health (imaging visit tabular data, FO):
    - BMI (21001.2 TD)
    - smoking (20116.2 TD)
        - Pre-processed into previous and current vs never at imaging
    - before imaging "First Occurrence" of common conditions (neurology, cardiovascular, malignancy)
        - G40-G41 epilepsy (131048) (FO)
        - G45-G46 cerebrovascular disease (131056, 131058) (FO)
        - I25 chronic ischaemic heart disease (131306) (FO)
        - Self-reported malignancies (134.2.0 TD)
            - Pre-processed into never vs 1 or more cancers before imaging OR baseline
- Mental health (MHFU, imaging visit tabular data)
    - substance use
        - alcohol consumption frequency
            - Pre-processed as units per week across 1568.2.0 / 1578.2.0 / 1588.2.0 / 1598.2.0 / 5364.0.0 / 1608.2.0 (one unit equals 10ml or 8g of pure alcohol)

Health exclusions (participants excluded if occur at or within 2 years after imaging)

- "First Occurrence" (FO) of dementia, degenerative and demyelinating disorders (G30-G37 Alzheimer's disease, degenerative disorders, demyelinating disorders (131036; 131038; 131040; 131042; 131044; 131046)(FO)
- G10 Huntington's disease (131012) (FO)
- G20 Parkinson's disease (131022) (FO)
- I60 subarachnoid haemorrhage (131360), I61 intracerebral haemorrhage (131362), I63 cerebral infarction (131366) (FO)

Prior checks to confirm validity of imputation

1. Load cognitive dataset & transfer across age and sex

```
z_cog_trial <- readRDS("~/OneDrive - Nexus365/Postdoc_funding/GoB_work/UKB/Inputs_derived/cognition_standardised_scores_fullUKBsample_Feb24.rds")

preproc_demo_slimmed<- readRDS("Inputs_derived/UKB_remitteddep_cog_demographics_preprocessed_Feb24.rds")

#z_cog_trial <- merge(z_cog_trial, preproc_demo_slimmed[c("f.eid", "Sex", "Age_ass_V2")], by = "f.eid", all.x = TRUE)

z_cog_trial <- merge(z_cog_trial, preproc_demo_slimmed, by = "f.eid", all.x = TRUE)
```

2. Multiple imputation and trial modelling & then check SD across the imputed datasets

```
# Load necessary libraries / packages
library(mice)
library(pool)
library(dplyr)
library(nnet)
library(lme4)
## GROUP A (FO+HES)

# Step 1: Impute missing data
# Step 1.1: Remove rows with no data (all empty rows except f.eid/pt id)
z_cog_trial <- z_cog_trial[!apply(is.na(z_cog_trial[-1]), 1, all),]

# Check the filtered dataset (remove the "stop" line if it looks correct)
#stop("Check the filtered dataset")



# Step 1.2: Impute missing values
# Create a function to z-score a column
zscore <- function(x) {
```

```
  (x - mean(x, na.rm = TRUE)) / sd(x, na.rm = TRUE)
}

# Columns to be imputed
cols_to_impute <- c(
  "reaction_V2_inverse_normal_scores",
  "correct_symbol_digit_matches",
  "fluid_intell_V2",
  "matrix_pattern_puzzles_solved",
  "numeric_cog_V2",
  "TMTB_MINUS_TMTA_V2_inverse_normal_scores",
  "correct_tower_arranging",
  "number_correct_paired_associates_V2_inverse_normal_scores"
)

# Columns to use for imputation
impute_columns <- cols_to_impute

# Convert the columns to numeric vectors - actually think all cog variables in raw data are raw anyway
#for (col in cols_to_impute) {
  #z_cog_trial[[col]] <- as.numeric(z_cog_trial[[col]])
#}

# Auxiliary variables to improve imputation (but not for imputing)
auxiliary_vars <- c("Sex", "Age_ass_V2")

# Create an imputation model (first iteration works without auxiliary variables, or can then add them directly into imputation below (hashtagged out if auxiliaries are in model itself as in iteration two)
#imputation_model <- mice(z_cog_trial[impute_columns],
                        #method = "pmm",
                        #m = 20, # Number of imputations
```

```
                    #maxit = 50)



# Convert f.eid columns in both datasets to character
z_cog_trial$f.eid <- as.character(z_cog_trial$f.eid)
preproc_demo_slimmed$f.eid <- as.character(preproc_demo_slimmed$f.eid)

# Convert data.table to data.frame
preproc_demo_slimmed_df <- as.data.frame(preproc_demo_slimmed)

# Perform a left join using dplyr
merged_data <- left_join(
  select(z_cog_trial, f.eid, all_of(impute_columns)),
  select(preproc_demo_slimmed_df, f.eid, Sex, Age_ass_V2),
  by = "f.eid"
)



# Ensure merged_data is a data frame
merged_data <- as.data.frame(merged_data)

# Specify which variables to impute
impute_vars <- c(
  "reaction_V2_inverse_normal_scores",
  "correct_symbol_digit_matches",
  "fluid_intell_V2",
  "matrix_pattern_puzzles_solved",
  "numeric_cog_V2",
  "TMTB_MINUS_TMTA_V2_inverse_normal_scores",
  "correct_tower_arranging",
  "number_correct_paired_associates_V2_inverse_normal_scores"
)
```

```
# Create an imputation model
imputation_model <- mice(
  data = merged_data,
  method = "pmm",
  m = 20,  # Number of imputations
  maxit = 50,
  impute = impute_vars,
  blockPredictor = list(
    c(1, 2, 3, 4, 5, 6, 7, 8),  # Predictor block for cognitive variables
    c(9, 10)  # Predictor block for auxiliary variables (Sex, Age_ass_V2)
  )
)
```

```
 iter imp variable
  1   1  correct_symbol_digit_matches  fluid_intell_V2  matrix_pattern_puzzles_solved numeric_cog_V2  correct_tower_arranging  Sex  Age_ass_V2
  1   2  correct_symbol_digit_matches  fluid_intell_V2  matrix_pattern_puzzles_solved numeric_cog_V2  correct_tower_arranging  Sex  Age_ass_V2
  1   3  correct_symbol_digit_matches  fluid_intell_V2  matrix_pattern_puzzles_solved numeric_cog_V2  correct_tower_arranging  Sex  Age_ass_V2
  1   4  correct_symbol_digit_matches  fluid_intell_V2  matrix_pattern_puzzles_solved numeric_cog_V2  correct_tower_arranging  Sex  Age_ass_V2
  1   5  correct_symbol_digit_matches  fluid_intell_V2  matrix_pattern_puzzles_solved numeric_cog_V2  correct_tower_arranging  Sex  Age_ass_V2
  1   6  correct_symbol_digit_matches  fluid_intell_V2  matrix_pattern_puzzles_solved numeric_cog_V2  correct_tower_arranging  Sex  Age_ass_V2
  1   7  correct_symbol_digit_matches  fluid_intell_V2  matrix_pattern_puzzles_solved numeric_cog_V2  correct_tower_arranging  Sex  Age_ass_V2
  1   8  correct_symbol_digit_matches  fluid_intell_V2  matrix_pattern_puzzles_solved numeric_cog_V2  correct_tower_arranging  Sex  Age_ass_V2
  1   9  correct_symbol_digit_matches  fluid_intell_V2  matrix_pattern_puzzles_solved numeric_cog_V2  correct_tower_arranging  Sex  Age_ass_V2
  1   10  correct_symbol_digit_matches  fluid_intell_V2  matrix_pattern_puzzles_solved numeric_cog_V2  correct_tower_arranging  Sex  Age_ass_V2
```

1 11 correct_symbol_digit_matches fluid_intell_V2 matrix_pattern_puzzles_solved numeric_cog_V2 correct_tower_arranging Sex Age_ass_V2

1 12 correct_symbol_digit_matches fluid_intell_V2 matrix_pattern_puzzles_solved numeric_cog_V2 correct_tower_arranging Sex Age_ass_V2

1 13 correct_symbol_digit_matches fluid_intell_V2 matrix_pattern_puzzles_solved numeric_cog_V2 correct_tower_arranging Sex Age_ass_V2

1 14 correct_symbol_digit_matches fluid_intell_V2 matrix_pattern_puzzles_solved numeric_cog_V2 correct_tower_arranging Sex Age_ass_V2

1 15 correct_symbol_digit_matches fluid_intell_V2 matrix_pattern_puzzles_solved numeric_cog_V2 correct_tower_arranging Sex Age_ass_V2

1 16 correct_symbol_digit_matches fluid_intell_V2 matrix_pattern_puzzles_solved numeric_cog_V2 correct_tower_arranging Sex Age_ass_V2

1 17 correct_symbol_digit_matches fluid_intell_V2 matrix_pattern_puzzles_solved numeric_cog_V2 correct_tower_arranging Sex Age_ass_V2

1 18 correct_symbol_digit_matches fluid_intell_V2 matrix_pattern_puzzles_solved numeric_cog_V2 correct_tower_arranging Sex Age_ass_V2

1 19 correct_symbol_digit_matches fluid_intell_V2 matrix_pattern_puzzles_solved numeric_cog_V2 correct_tower_arranging Sex Age_ass_V2

1 20 correct_symbol_digit_matches fluid_intell_V2 matrix_pattern_puzzles_solved numeric_cog_V2 correct_tower_arranging Sex Age_ass_V2

2 1 correct_symbol_digit_matches fluid_intell_V2 matrix_pattern_puzzles_solved numeric_cog_V2 correct_tower_arranging Sex Age_ass_V2

2 2 correct_symbol_digit_matches fluid_intell_V2 matrix_pattern_puzzles_solved numeric_cog_V2 correct_tower_arranging Sex Age_ass_V2

2 3 correct_symbol_digit_matches fluid_intell_V2 matrix_pattern_puzzles_solved numeric_cog_V2 correct_tower_arranging Sex Age_ass_V2

2 4 correct_symbol_digit_matches fluid_intell_V2 matrix_pattern_puzzles_solved numeric_cog_V2 correct_tower_arranging Sex Age_ass_V2

2 5 correct_symbol_digit_matches fluid_intell_V2 matrix_pattern_puzzles_solved numeric_cog_V2 correct_tower_arranging Sex Age_ass_V2

2 6 correct_symbol_digit_matches fluid_intell_V2 matrix_pattern_puzzles_solved numeric_cog_V2 correct_tower_arranging Sex Age_ass_V2

2 7 correct_symbol_digit_matches fluid_intell_V2 matrix_pattern_puzzles_solved numeric_cog_V2 correct_tower_arranging Sex Age_ass_V2

2 8 correct_symbol_digit_matches fluid_intell_V2 matrix_pattern_puzzles_solved numeric_cog_V2 correct_tower_arranging Sex Age_ass_V2

2 9 correct_symbol_digit_matches fluid_intell_V2 matrix_pattern_puzzles_solved numeric_cog_V2 correct_tower_arranging Sex Age_ass_V2

2 10 correct_symbol_digit_matches fluid_intell_V2 matrix_pattern_puzzles_solved numeric_cog_V2 correct_tower_arranging Sex Age_ass_V2

2 11 correct_symbol_digit_matches fluid_intell_V2 matrix_pattern_puzzles_solved numeric_cog_V2 correct_tower_arranging Sex Age_ass_V2

2 12 correct_symbol_digit_matches fluid_intell_V2 matrix_pattern_puzzles_solved numeric_cog_V2 correct_tower_arranging Sex Age_ass_V2

2 13 correct_symbol_digit_matches fluid_intell_V2 matrix_pattern_puzzles_solved numeric_cog_V2 correct_tower_arranging Sex Age_ass_V2

2 14 correct_symbol_digit_matches fluid_intell_V2 matrix_pattern_puzzles_solved numeric_cog_V2 correct_tower_arranging Sex Age_ass_V2

2 15 correct_symbol_digit_matches fluid_intell_V2 matrix_pattern_puzzles_solved numeric_cog_V2 correct_tower_arranging Sex Age_ass_V2

2 16 correct_symbol_digit_matches fluid_intell_V2 matrix_pattern_puzzles_solved numeric_cog_V2 correct_tower_arranging Sex Age_ass_V2

2 17 correct_symbol_digit_matches fluid_intell_V2 matrix_pattern_puzzles_solved numeric_cog_V2 correct_tower_arranging Sex Age_ass_V2

2 18 correct_symbol_digit_matches fluid_intell_V2 matrix_pattern_puzzles_solved numeric_cog_V2 correct_tower_arranging Sex Age_ass_V2

2 19 correct_symbol_digit_matches fluid_intell_V2 matrix_pattern_puzzles_solved numeric_cog_V2 correct_tower_arranging Sex Age_ass_V2

2 20 correct_symbol_digit_matches fluid_intell_V2 matrix_pattern_puzzles_solved numeric_cog_V2 correct_tower_arranging Sex Age_ass_V2

TRUNCATED FOR SPACE

50 1 correct_symbol_digit_matches fluid_intell_V2 matrix_pattern_puzzles_solved numeric_cog_V2 correct_tower_arranging Sex Age_ass_V2

50 2 correct_symbol_digit_matches fluid_intell_V2 matrix_pattern_puzzles_solved numeric_cog_V2 correct_tower_arranging Sex Age_ass_V2

50 3 correct_symbol_digit_matches fluid_intell_V2 matrix_pattern_puzzles_solved numeric_cog_V2 correct_tower_arranging Sex Age_ass_V2

50 4 correct_symbol_digit_matches fluid_intell_V2 matrix_pattern_puzzles_solved numeric_cog_V2 correct_tower_arranging Sex Age_ass_V2

50 5 correct_symbol_digit_matches fluid_intell_V2 matrix_pattern_puzzles_solved numeric_cog_V2 correct_tower_arranging Sex Age_ass_V2

50 6 correct_symbol_digit_matches fluid_intell_V2 matrix_pattern_puzzles_solved numeric_cog_V2 correct_tower_arranging Sex Age_ass_V2

50 7 correct_symbol_digit_matches fluid_intell_V2 matrix_pattern_puzzles_solved numeric_cog_V2 correct_tower_arranging Sex Age_ass_V2

50 8 correct_symbol_digit_matches fluid_intell_V2 matrix_pattern_puzzles_solved numeric_cog_V2 correct_tower_arranging Sex Age_ass_V2

50 9 correct_symbol_digit_matches fluid_intell_V2 matrix_pattern_puzzles_solved numeric_cog_V2 correct_tower_arranging Sex Age_ass_V2

```
  50   10  correct_symbol_digit_matches  fluid_intell_V2  matrix_pattern_puzzles_solved  numeric_cog_V2  correct_tower_arranging  Sex  Age_ass_V2
  50   11  correct_symbol_digit_matches  fluid_intell_V2  matrix_pattern_puzzles_solved  numeric_cog_V2  correct_tower_arranging  Sex  Age_ass_V2
  50   12  correct_symbol_digit_matches  fluid_intell_V2  matrix_pattern_puzzles_solved  numeric_cog_V2  correct_tower_arranging  Sex  Age_ass_V2
  50   13  correct_symbol_digit_matches  fluid_intell_V2  matrix_pattern_puzzles_solved  numeric_cog_V2  correct_tower_arranging  Sex  Age_ass_V2
  50   14  correct_symbol_digit_matches  fluid_intell_V2  matrix_pattern_puzzles_solved  numeric_cog_V2  correct_tower_arranging  Sex  Age_ass_V2
  50   15  correct_symbol_digit_matches  fluid_intell_V2  matrix_pattern_puzzles_solved  numeric_cog_V2  correct_tower_arranging  Sex  Age_ass_V2
  50   16  correct_symbol_digit_matches  fluid_intell_V2  matrix_pattern_puzzles_solved  numeric_cog_V2  correct_tower_arranging  Sex  Age_ass_V2
  50   17  correct_symbol_digit_matches  fluid_intell_V2  matrix_pattern_puzzles_solved  numeric_cog_V2  correct_tower_arranging  Sex  Age_ass_V2
  50   18  correct_symbol_digit_matches  fluid_intell_V2  matrix_pattern_puzzles_solved  numeric_cog_V2  correct_tower_arranging  Sex  Age_ass_V2
  50   19  correct_symbol_digit_matches  fluid_intell_V2  matrix_pattern_puzzles_solved  numeric_cog_V2  correct_tower_arranging  Sex  Age_ass_V2
  50   20  correct_symbol_digit_matches  fluid_intell_V2  matrix_pattern_puzzles_solved  numeric_cog_V2  correct_tower_arranging  Sex  Age_ass_V2
Warning: Number of logged events: 1
# Perform imputation
imputed_data <- complete(imputation_model)


# Create a list to store the individual imputed datasets
imputed_datasets <- list()


# Access each imputed dataset and store them in the list
for (i in 1:20) {
  # Perform imputation for the current dataset
  imputed_data <- complete(imputation_model, action = i)


  # Add the 'f.eid' column to the imputed dataset
  #imputed_data$f.eid <- z_cog_trial$f.eid
```

```
  # Add auxiliary variables (age and gender) to the imputed dataset
  #imputed_data$Sex <- z_cog_trial$Sex
  #imputed_data$Age_ass_V2 <- z_cog_trial$Age_ass_V2

  # Add the imputed dataset to the list
  imputed_datasets[[i]] <- imputed_data
}



# Step 1.3: Recalculate the derived z-scores
# Create a list to store the processed imputed datasets
processed_imputed_datasets <- list()

# Loop through each imputed dataset
for (i in 1:20) {
  imputed_data <- imputed_datasets[[i]]

  # Recalculate the derived z-scores
  imputed_data$processing_speed <- rowMeans(
    imputed_data[c("reaction_V2_inverse_normal_scores", "correct_symbol_digit_matches")],
    na.rm = TRUE
  )
  imputed_data$processing_speed_zscore <- scale(imputed_data$processing_speed)

  imputed_data$reasoning <- rowMeans(
    imputed_data[c("fluid_intell_V2", "matrix_pattern_puzzles_solved")],
    na.rm = TRUE
  )
  imputed_data$reasoning_zscore <- scale(imputed_data$reasoning)

  imputed_data$executive_function <- rowMeans(
```

```
    imputed_data[c("numeric_cog_V2", "TMTB_MINUS_TMTA_V2_inverse_normal_scores", "correct_tower_arranging")],
    na.rm = TRUE
  )
  imputed_data$executive_function_zscore <- scale(imputed_data$executive_function)



  # Step 1.4: Set memory_zscore as number_correct_paired_associates_V2_zscore
  imputed_data$memory_zscore <- scale(imputed_data$number_correct_paired_associates_V2_inverse_normal_scores)



  # Step 1.5: Calculate the row means of all domains to give a summed score
  imputed_data$summed_score <- rowMeans(imputed_data[, c("processing_speed", "reasoning", "executive_function", "number_correct_paired_associates_V2_inverse_normal_scores")])
  # Create z score of summed score
  imputed_data$summed_zscores <- scale(imputed_data$summed_score)

  # Store the processed imputed dataset in the list
  processed_imputed_datasets[[i]] <- imputed_data
}

saveRDS(processed_imputed_datasets, file = "Inputs_derived/processed_imputed_datasets_Feb24.rds")
# Extract the lengths of each imputed dataset
dataset_lengths <- sapply(processed_imputed_datasets, function(dataset) nrow(dataset))

# Print the lengths
print(dataset_lengths)
 [1] 502226 502226 502226 502226 502226 502226 502226 502226 502226 502226
502226 502226 502226 502226 502226 502226 502226 502226 502226 502226
# Check if all lengths are the same
all_lengths_same <- all(dataset_lengths == dataset_lengths[1])
```

```
if (all_lengths_same) {
  cat("All imputed datasets have the same length.\n")
} else {
  cat("Imputed datasets have different lengths.\n")
}
All imputed datasets have the same length.
# Get list of f.eids from the imputed data (first dataset)
pt_ids_from_first_imputed_dataset <- processed_imputed_datasets[[1]]$f.eid

# Need this for later 2_match_cohorts
```

Supplementary Results

Supplementary Figure 1: Flow diagram

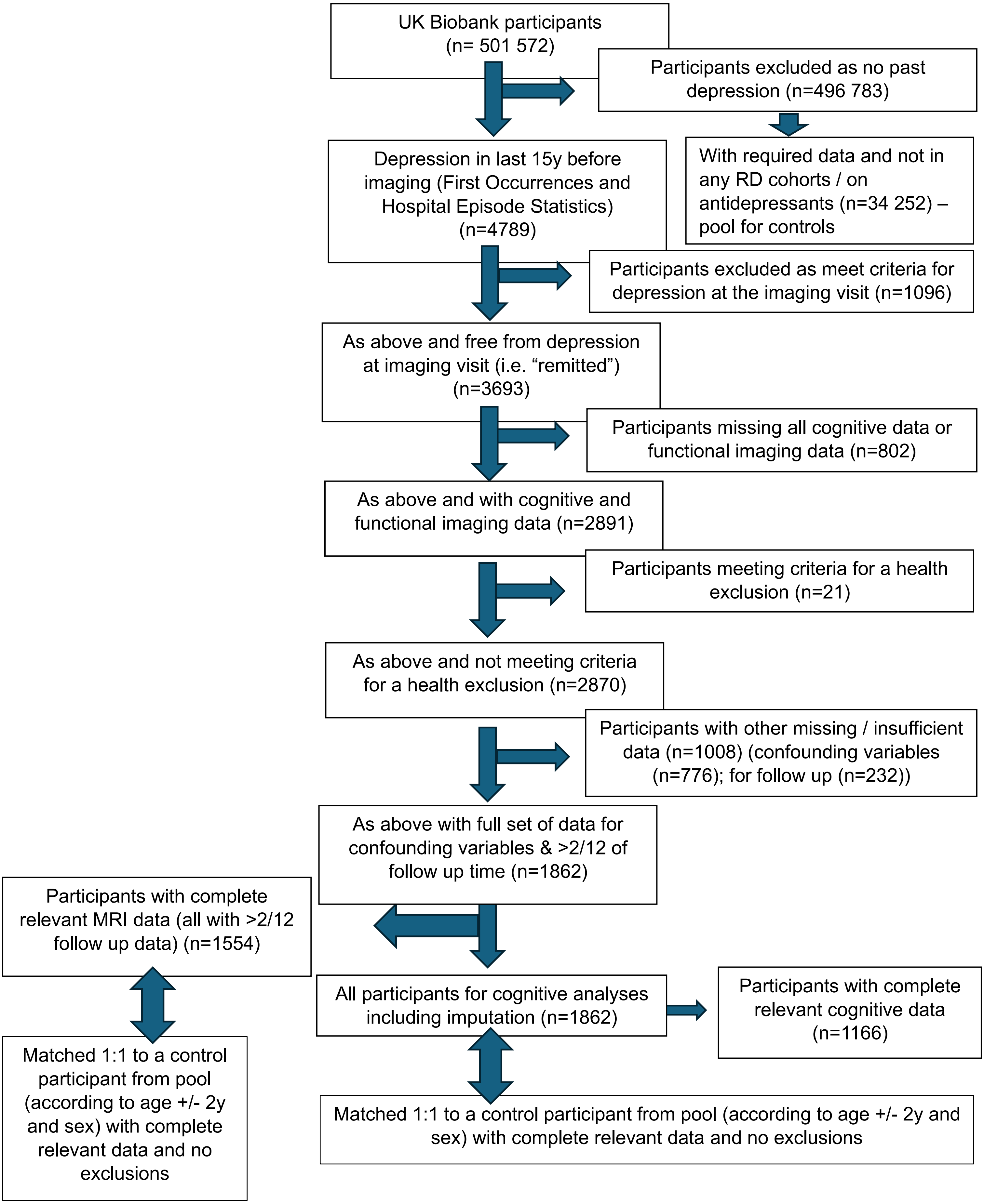

Supplementary Table 1(a): Comparison between those with complete and incomplete cognitive data in the FO+HES RD / control cohorts for age and sex

Age:

completer <chr>	group <chr>	n <int>	mean_age <dbl>	sd_age <dbl>
Completers	Controls	1129	52.36	7.03
Completers	Depression	1166	52.40	7.04
Non-completers	Controls	733	53.12	7.27
Non-completers	Depression	696	53.29	7.24

Sex:

Group <chr>	Males <int>	Females <int>
Completers/Controls	677	452
Completers/Depression	710	456
Non-comp/Controls	430	303
Non-comp/Depression	397	299

Antidepressant use at imaging (Remitted Depression group only):

	No <int>	Yes <int>
Completers	748	418
Non-completers	465	231

Supplementary Table 1(b): Characteristics at baseline and health factors at imaging of participants with incomplete data for control variables and follow-up time

Characteristic	Matched control cohort (2870)	Primary RD cohort (2870)
Age (years)	52.3 (7.33)	52.4 (7.20)
Sex (%): Female; Male	1833 (63.9); 1037 (36.1)	1833 (63.9); 1037 (36.1)
Townsend Deprivation Index (%)		
1 (= least deprived)	1280 (44.6)	1195 (41.6)
2	1046 (36.4)	1109 (38.6)
3	405 (14.1)	409 (14.3)
4	126 (4.39)	145 (5.05)
5 (= most deprived)	8 (0.29)	10 (0.35)
Missing	5 (0.17)	2 (0.07)
Postsecondary Education (%)		
Post university	1290 (44.9)	1252 (43.6)
A levels	383 (13.3)	384 (13.4)
O levels	586 (20.4)	610 (21.3)
CSEs	143 (4.98)	147 (5.12)
NVQ / HND	139 (4.84)	141 (4.91)
Other professional	127 (4.43)	141 (4.91)
None of the above	148 (5.15)	149 (5.19)
Missing	54 (1.88)	46 (1.60)
Ethnicity (%)		
White	2769 (96.5)	2783 (97.0)
Black	27 (0.94)	15 (0.52)
Other ethnicity groups	69 (2.40)	61 (2.13)
Missing	5 (0.17)	11 (0.40)
Health factors at imaging assessment		
Never smoker	1855 (65.3)	1690 (59.1)
Alcohol intake (mean units / week)	16.4 (14.2)	16.1 (15.3)
Body mass index (BMI kg / m2) at imaging	26.3 (4.44)	27.6 (4.97)

Existing conditions at imaging assessment		
Epilepsy	26 (0.91)	33 (1.15)
Cerebrovascular disease	39 (1.36)	49 (1.71)
Ischaemic heart disease	89 (3.10)	171 (5.96)
Previous cancer diagnosis	332 (11.6)	430 (15.0)
Depression factors		
Antidepressant use at imaging (%)	0 (0)	981 (34.2)

Supplementary Table 1(c): Characteristics at baseline and health factors at imaging of participants with complete MRI data

Characteristic	Matched control cohort (1554)	Primary RD cohort (1554)
Age (years)	52.6 (7.34)	52.9 (7.19)
Sex (%): Female; Male	923 (59.4); 631 (40.6)	923 (59.4); 631 (40.6)
Townsend Deprivation Index (%)		
1 (= least deprived)	721 (46.5)	672 (43.2)
2	567 (36.4)	602 (38.7)
3	209 (13.4)	207 (13.3)
4 + 5 = most deprived (conflated due to small numbers)	57 (3.7)	73 (4.8)
Postsecondary Education (%)		
Post university	766 (49.2)	733 (47.2)
A levels	206 (13.3)	213 (13.4)
O levels	311 (20.1)	317 (20.4)
CSEs	80 (5.15)	78 (5.02)
NVQ / HND	73 (4.70)	72 (4.63)
Other professional	58 (3.73)	71 (4.57)
None of the above	60 (3.86)	70 (4.50)
Ethnicity (%)		
White	1520 (97.8)	1532 (98.6)
Other ethnicity groups (conflated due to small numbers)	34 (2.2)	22 (1.4)
Health factors at imaging assessment		
Never smoker	998 (64.2)	877 (56.4)
Alcohol intake (mean units / week)	16.5 (14.0)	16.2 (15.1)
Body mass index (BMI kg / m2) at imaging	26.1 (4.28)	27.2 (4.68)
Existing conditions at imaging assessment		
Epilepsy	6 (0.39)	15 (0.97)
Cerebrovascular disease	23 (1.48)	36 (2.31)

Ischaemic heart disease	60 (3.86)	109 (7.01)
Previous cancer diagnosis	170 (10.9)	231 (14.9)
Depression factors		
Antidepressant use at imaging (%)	0 (0)	537 (34.6)

Supplementary Table 2: Comparison between remitted depressed cohort with complete data for control variables (n=1862) and matched control cohort (n=1862) for FO+HES cohort

Characteristic / health factor	Estimate	P value
Ethnicity	8.40 (chi-sq)	**0.02**
Education	5.49 (chi-sq)	0.51
Deprivation (Townsend Index)	11.4 (chi-sq)	**0.02**
BMI	91.2 (F)	**<0.001**
Smoking	21.3 (chi-sq)	**<0.001**
Epilepsy	3.13 (chi-sq)	0.08
Cerebrovascular disease	4.65 (chi-sq)	**0.03**
Ischaemic heart disease	28.3 (chi-sq)	**<0.001**
Previous cancer	12.4 (chi-sq)	**<0.001**
Alcohol	1.58 (F)	0.20

Supplementary Table 3: Behavioural cognitive functioning at imaging (without imputation)

Summed scores for each cognitive domain and the overall composite measure combining all domains (scores below shown are z scores).

Tasks included as follows per domain: processing speed (reaction time, symbol digit substitution), reasoning (fluid intelligence, matrix pattern puzzles), executive function (digit span, TMT B-A, tower arranging), memory (paired associate learning).

Group <dbl>	Statistic <chr>	summed_zscores <dbl>	processing_speed <dbl>	reasoning <dbl>	executive_function <dbl>	memory <dbl>
0	Mean	0.15934971	0.19292264	0.08297103	0.12328060	−1.1043430
0	Median	0.17041068	0.22866891	0.19389773	0.10291115	−1.3575327
0	SD	0.92467765	0.93093236	0.97818621	0.99617558	1.0080718
1	Mean	0.06452471	0.06735194	0.07634521	0.04896192	−1.1178133
1	Median	0.09898357	0.10735936	0.19389773	0.08588481	−1.3575327
1	SD	0.97580334	0.99185223	0.96630991	0.99163546	0.9827933

Group 0 = Controls; Group 1 = Remitted depressed cohort (FO+HES)

Supplementary Table 4(a): Lifetime of depressed periods (self-report; f.20442 from the 2016-2017 online follow-up) for the FO+HES remitted depressed (RD) cohort

Lifetime number of depressed periods	Number of RD participants
1	337
2	114
3	166
4	56
5	46
6	31
7	5
8	8
9	3
10+	290
Total	1056

806 (43%) were missing data for this variable.

Supplementary Table 4(b): Anxiety Disorder (First Occurrences ICD-110 F41) for the FO+HES remitted depressed (RD) and control cohorts

**Group**	**Number of F41 FO total & prior to imaging (%)**
Controls	51; 46 (3%)
Remitted Depression	677; 609 (33%)

Supplementary Table 4(c): Sources of longitudinal data for future depressive episodes (n; %)

For participants with no previous episode of depression (n=240):

Hospital Episode Statistics	Mental Health Follow Up (record of age at last depression later than age at imaging)	Mental Health Follow Up (current PHQ-9>/=10)	Online Follow Up (self-report of recent depression)
3 (1.3%)	12 (5%)	210 (88%)	239 (97%)

For participants with remitted depression (n=623):

Hospital Episode Statistics	Mental Health Follow Up (record of age at last depression later than age at imaging)	Mental Health Follow Up (current PHQ-9>/=10)	Online Follow Up (self-report of recent depression)
272 (44%)	501 (80%)	500 (80%)	598 (96.0%)

NB. Some participants have > one simultaneous report of depression in follow-up period

Supplementary Figure 2(a): Adjusted hazard ratios from proportional hazards cox model for Figure 1(B)

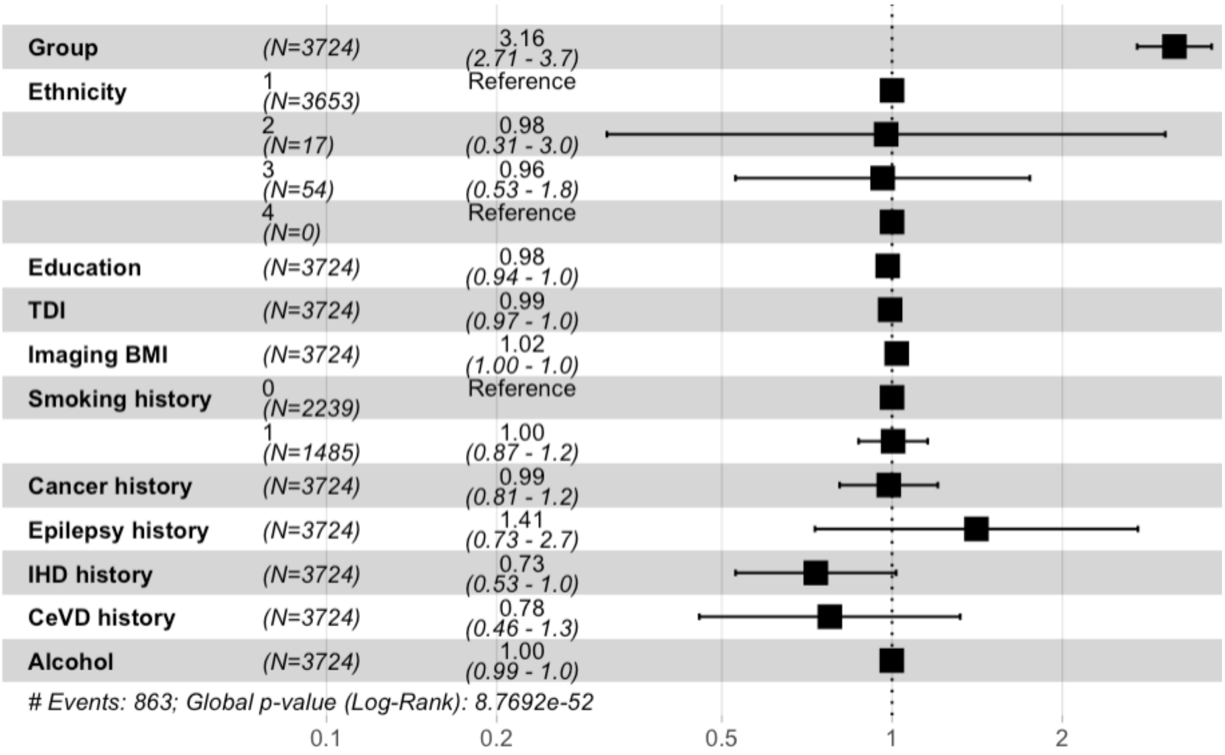

TDI = Townsend Deprivation Index; IHD = Ischaemic Heart Disease; CeVD = Cerebrovascular Disease

Supplementary Figure 2(b): Kaplan-Meier survival curve as Figure 1(B) (truncated to 2 years follow up)

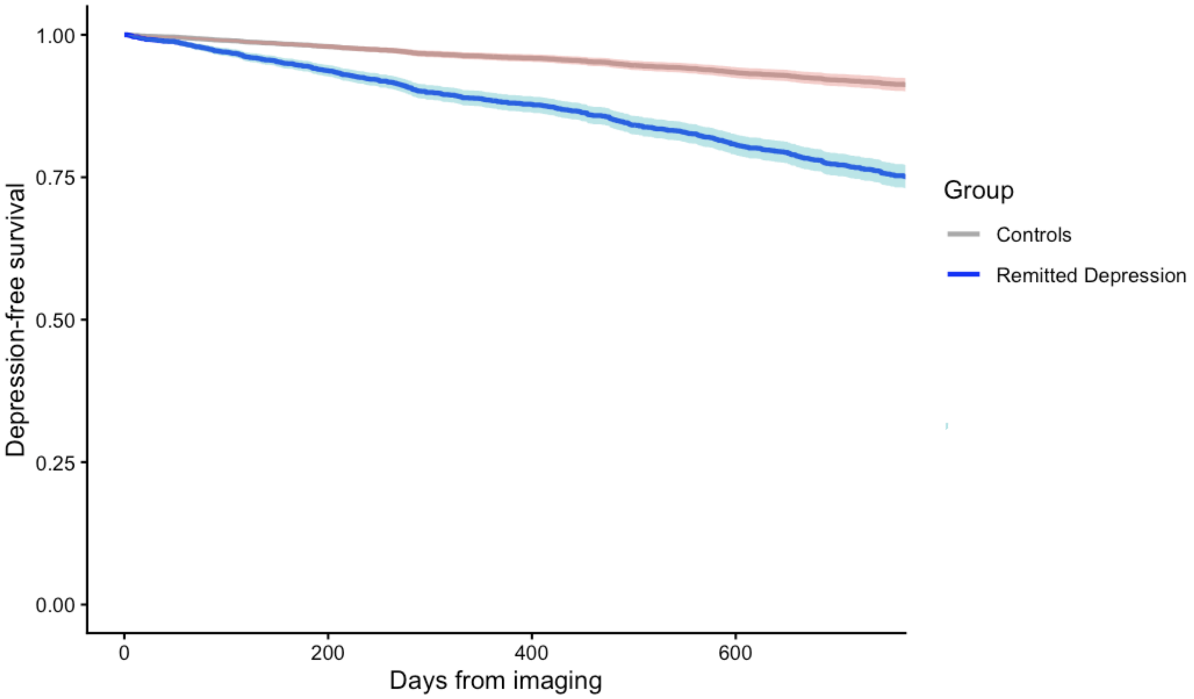

Supplementary Table 5: Proportional hazards for Figure 1(B)

	Chisq	P value
Group	0.003	0.95
Ethnicity	3.09	0.21
Education	0.19	0.66
TDI	1.68	0.20
Imaging BMI	6.01	0.02
Smoking history	3.95	0.05
Cancer history	1.41	0.23
Epilepsy history	0.16	0.69
IHD history	2.72	0.10
CeVD history	0.02	0.88
Alcohol (units/wk)	0.51	0.48
GLOBAL	20.47	0.07

TDI = Townsend Deprivation Index; IHD = Ischaemic Heart Disease; CeVD = Cerebrovascular Disease

Supplementary Table 6: Risk of future depressive episodes and impact of composite cognitive scores at imaging visit

Analyses		Beta (estimate)	Standard error	Z score	P value
Main model	*Effect of composite cognitive score*	-0.257	0.114	-2.23	0.022*
	*Composite cognitive score*group*	0.470	0.136	3.42	0.0006*
Model without group	*Effect of composite cognition score*	0.025	0.067	0.371	0.711
Main model with imputation	*Effect of composite cognitive score*	-0.121	0.079	-1.923	0.052
	*Composite cognitive score*group*	0.285	0.096	2.95	0.003*

Supplementary Figure 3: Future depression risk relating to cognition scores at imaging visit for all comers (model without group)

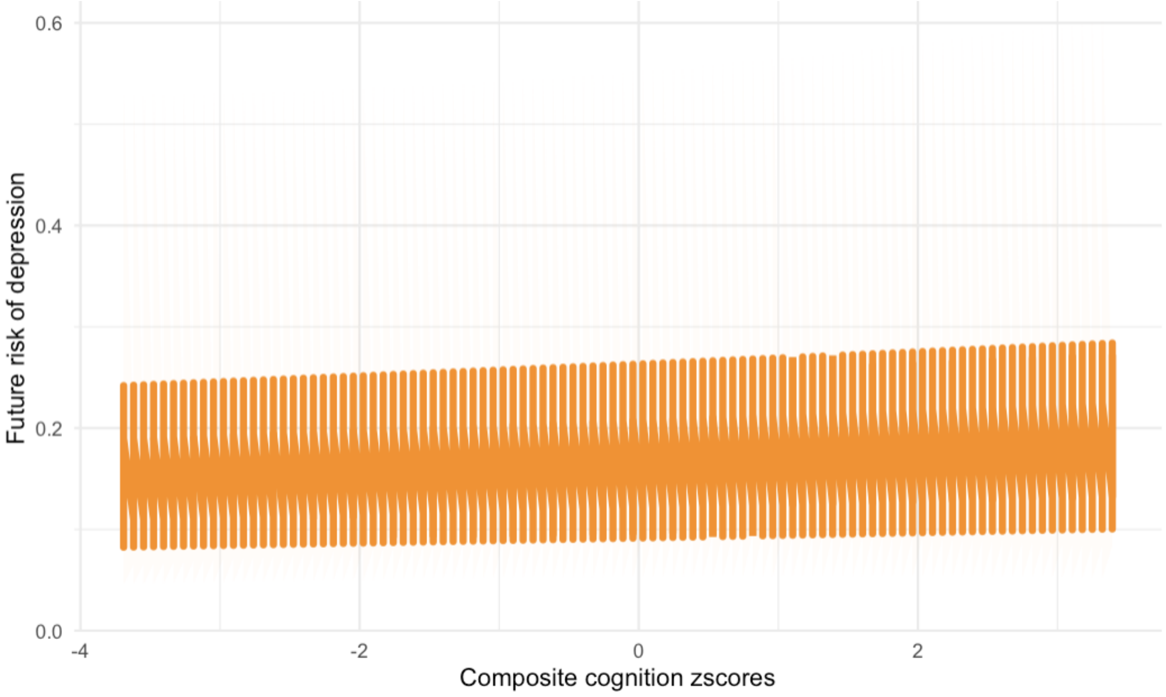

Future depression expressed as risk </=1. Cognitive scores expressed as a z score. Confidence intervals around the mean risk shown. (A) composite score across all dimensions; (B) memory scores (based on paired associate learning scores) (B) executive functioning scores (based on digit span, TMT B-A, tower arranging scores); (C) reasoning scores (based on fluid intelligence, matrix pattern puzzles scores); (D) processing speed scores (based on reaction time, symbol digit substitution scores)

Supplementary Table 7(a): Risk of future depressive episodes and impact of memory score (n=3724; RD:controls=1862:1862)

Analyses		Beta (estimate)	Standard error	Z score	P value
Main model	*Effect of memory score*	-0.41	0.077	-5.30	<0.001*
	*Memory score*group*	0.194	0.088	2.138	0.03*
Model without group	*Effect of memory scores*	-0.285	0.052	-5.50	<0.001*

Supplementary Table 7(b): Risk of future depressive episodes and impact of executive function (EF) score (n=2310; RD:controls=1174:1136)

Analyses		Beta (estimate)	Standard error	Z score	P value
Main model	*Effect of EF score*	-0.125	0.104	-1.196	0.23
	*EF score*group*	0.243	0.129	1.900	0.05(*)
Model without group	*Effect of EF score*	0.013	0.061	0.217	0.83

Supplementary Table 7(c): Risk of future depressive episodes and impact of reasoning score (n=2617; RD:controls=1315:1302)

Analyses		Beta (estimate)	Standard error	Z score	P value
Main model	*Effect of reasoning score*	-0.1812	0.110	-1.642	0.10
	*Reasoning score*group*	0.37	0.132	2.804	0.005*
Model without group	*Effect of reasoning score*	-0.074	0.066	1.118	0.26

Supplementary Table 7(d): Risk of future depressive episodes and impact of processing speed (PS) score (n=2639; RD:controls=1326:1313)

Analyses		Beta (estimate)	Standard error	Z score	P value
Main model	*Effect of PS score*	-0.247	0.107	-2.31	0.02*
	*PS score*group*	0.408	0.130	3.139	0.002*
Model without group	*Effect of PS score*	-0.012	0.060	-0.192	0.85

Supplementary Table 8: Risk of future depressive episodes and impact of individual cognitive tasks

Analyses (all main model)		Beta (estimate)	Standard error	Z score	P value
TMTB-A (time; inverse)	*Effect of task measure*	0.338	0.072	4.71	<0.001*
	*Task measure*group*	-0.071	0.082	-0.86	0.39
Reaction time (time; inverse)	*Effect of task measure*	0.098	0.121	0.81	0.42
	*Task measure*group*	-0.214	0.148	-1.40	0.16
Digit symbol matches (number correct)	*Effect of task measure*	-0.249	0.107	-2.33	0.02*
	*Task measure*group*	0.041	0.130	3.13	0.002*
Fluid intelligence (score)	*Effect of task measure*	-0.015	0.0802	-0.19	0.85
	*Task measure*group*	0.156	0.097	1.61	0.11
Matrix pattern puzzles (number solved correctly)	*Effect of task measure*	-0.090	0.104	-0.87	0.38
	*Task measure*group*	0.310	0.129	2.41	0.02*
Numerical cognition (score)	*Effect of task measure*	-0.061	0.099	-0.61	0.54
	*Task measure*group*	0.135	0.124	1.01	0.31
Tower arranging (number correct)	*Effect of task measure*	-0.107	0.099	-1.08	0.28
	*Task measure*group*	0.202	0.124	1.63	0.10
Paired associates (number correct)	*Effect of task measure*	-0.404	0.077	-5.25	<0.001*
	*Task measure*group*	0.188	0.088	2.14	0.03*

Supplementary Figure 4: Future depression risk relating to cognition scores at imaging visit for individual tasks

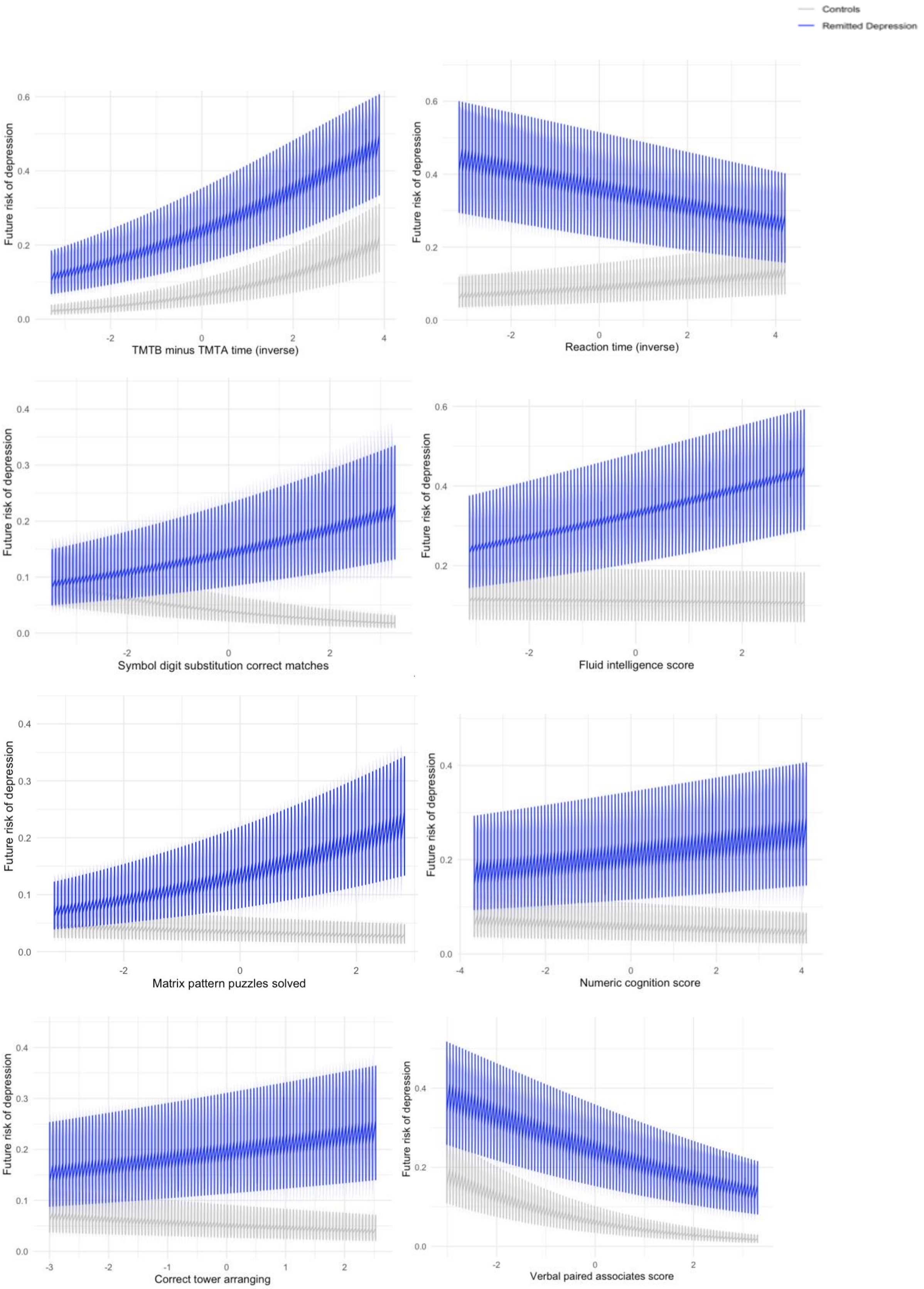

Future depression expressed as risk </=1. Cognitive scores expressed as a z score. Confidence intervals around the mean risk shown.

Results from Sensitivity Analyses for Primary analyses (using FO+HES cohort)

(i) **Excluding those pts who develop neurodegenerative disease or dementia within 10 years after imaging**

No additional participants excluded compared to developing neurodegenerative disease or dementia within two years.

(ii) **Excluding those pts who had cerebrovascular disease at imaging**

90 additional participants excluded from FO+HES cohort when conducted as complete cases for control variables (N=3634; 1:1 RD:control cohorts). Similar to main analyses, 219 (12%) in control cohort developed depression after imaging compared to 609 (33%) in the RD cohort.

(iii) **Using an additional control for those pts with close family history of depression**

291 (15.6%) RD participants had a close family history of depression.

(iv) **Dividing cohorts into 64 years and less versus 65 and over**

In the RD cohort, 1104 participants were 64 years and less, and 758 were 65 and over.

For the <65y: 164 (15%) in control cohort developed depression after imaging compared to 433 (39%) in the RD cohort

For 65+: 76 (10%) in control cohort developed depression after imaging compared to 190 (25%) in the RD cohort.

Odds of future depression for control participants in <65: 0.14

Odds of future depression for RD participants in <65: 0.53

Odds of future depression for control participants in >/=65: 0.099

Odds of future depression for RD participants in >/=65: 0.29

Chi squared results between the cohort splits: Xsq = 0.075, p value 0.78

Supplementary Table 9: Sensitivity analyses for primary outcome

**Analyses**		**Total n (RD: Control)**		**Beta (estimate)**	**Standard error**	**Z score**	**P value**
No neurodegenerative disease or dementia in 10y after imaging		*3724 (1862: 1862)*		Same as main analyses (no additional pts excluded)			
No cerebrovascular disease present at imaging visit	*Model without group*	*3634 (1817: 1817)*	*Effect of composite cognition score*	0.013	0.065	1.75	0.080
	Main model		*Effect of composite cognitive score*	-0.076	0.107	-0.714	0.479
	Main model		*Composite cognitive score*group*	0.305	0.131	2.33	0.0199*
Additionally controlling for close family history of depression	*Model without group*	*3724 (1862: 1862)*	*Effect of composite cognition score*	0.0172	0.067	0.257	0.798
	Main model		*Effect of composite cognitive score*	-0.262	0.114	-2.30	0.022*
	Main model		*Composite cognitive score*group*	0.463	0.137	3.387	0.0007*
Only age 64 or less	*Main model*	*2208 (1104: 1104)*	*Effect of composite cognitive score*	-0.291	0.147	-1.97	0.0481*
	Main model		*Composite cognitive score*group*	0.441	0.180	2.45	0.0136*
Only age 65 or more	*Main model*	*1516 (758: 758)*	*Effect of composite cognitive score*	-0.209	0.206	-1.02	0.310
	Main model		*Composite cognitive score*group*	0.494	0.248	2.00	0.046*

Supplementary Figure 5: Future depression risk relating to composite cognition scores at imaging in the primary cohort: (A) 64 and under subset; (B) 65 and over subset

(A)

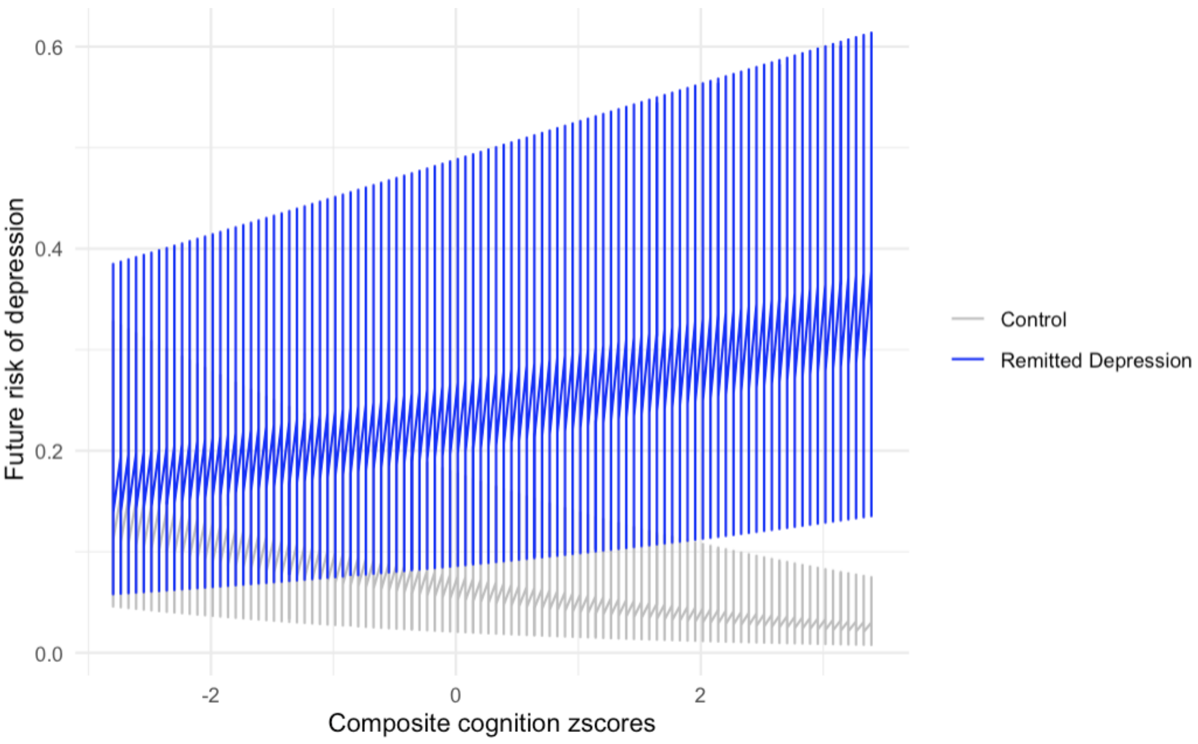

(B)

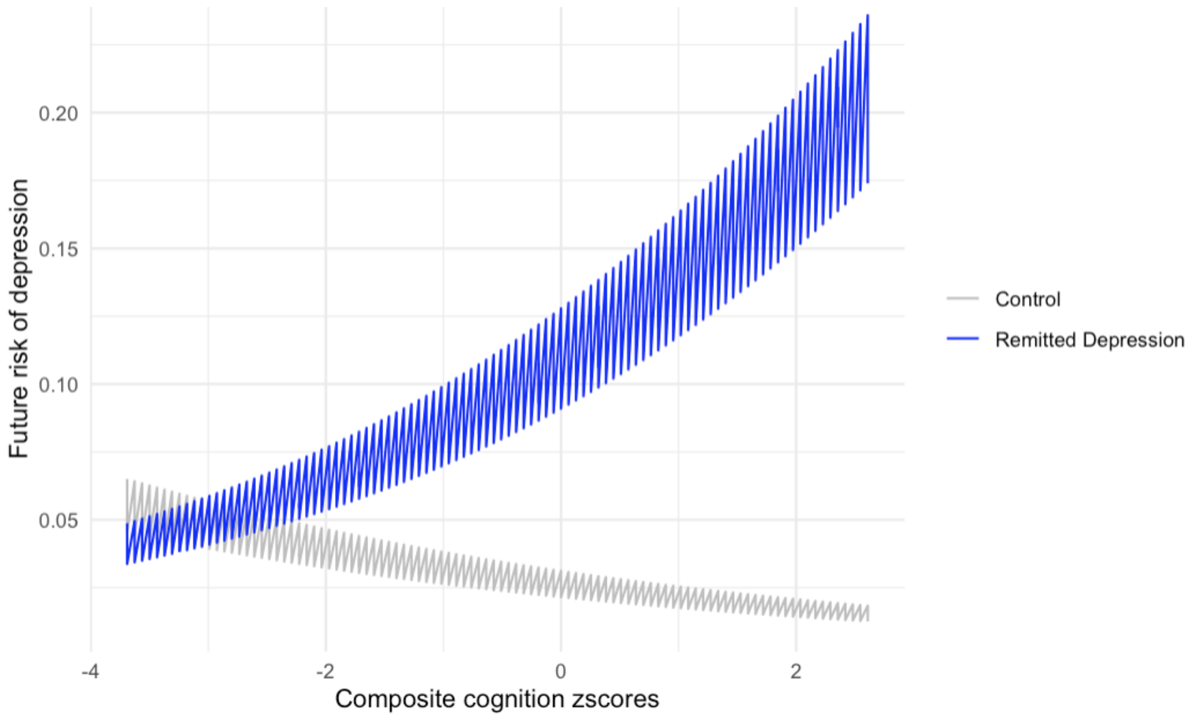

Supplementary Figure 6(a): Future depression risk relating to composite cognition scores at imaging in the primary cohort when Townsend Deprivation Index (TDI) included as an interaction term in the model

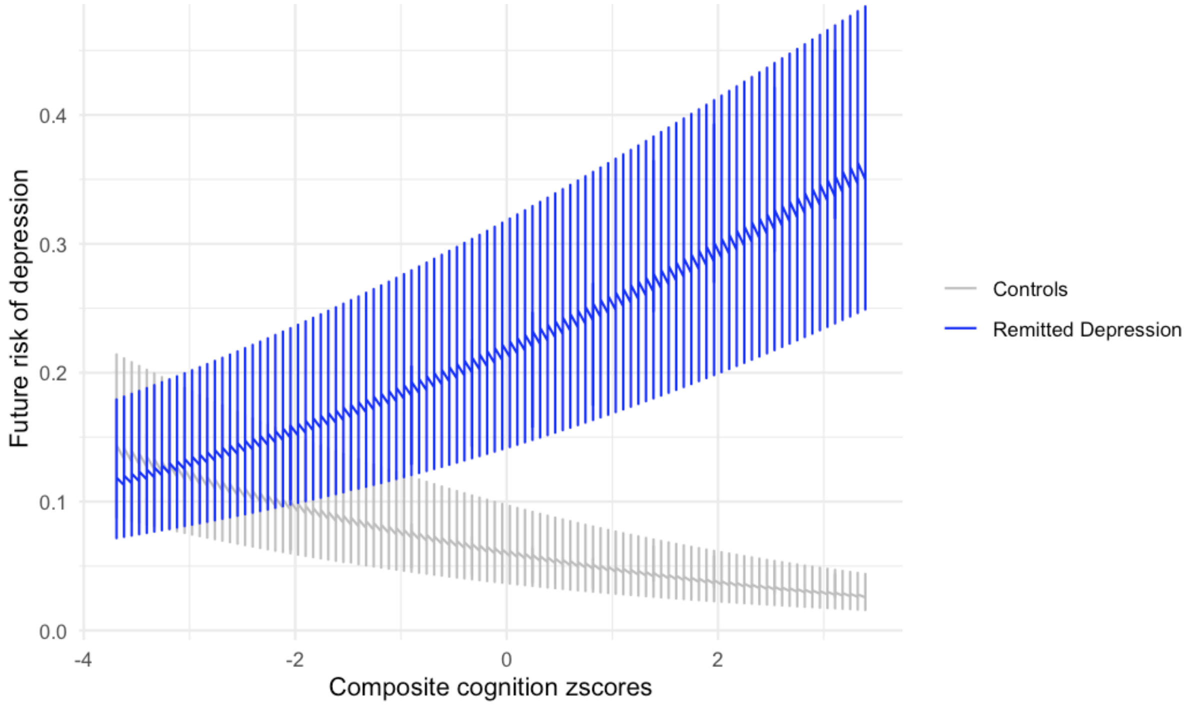

Supplementary Figure 6(b): Mean composite cognition by Townsend Deprivation Index (TDI) quintile and group (see Table 1 for n)

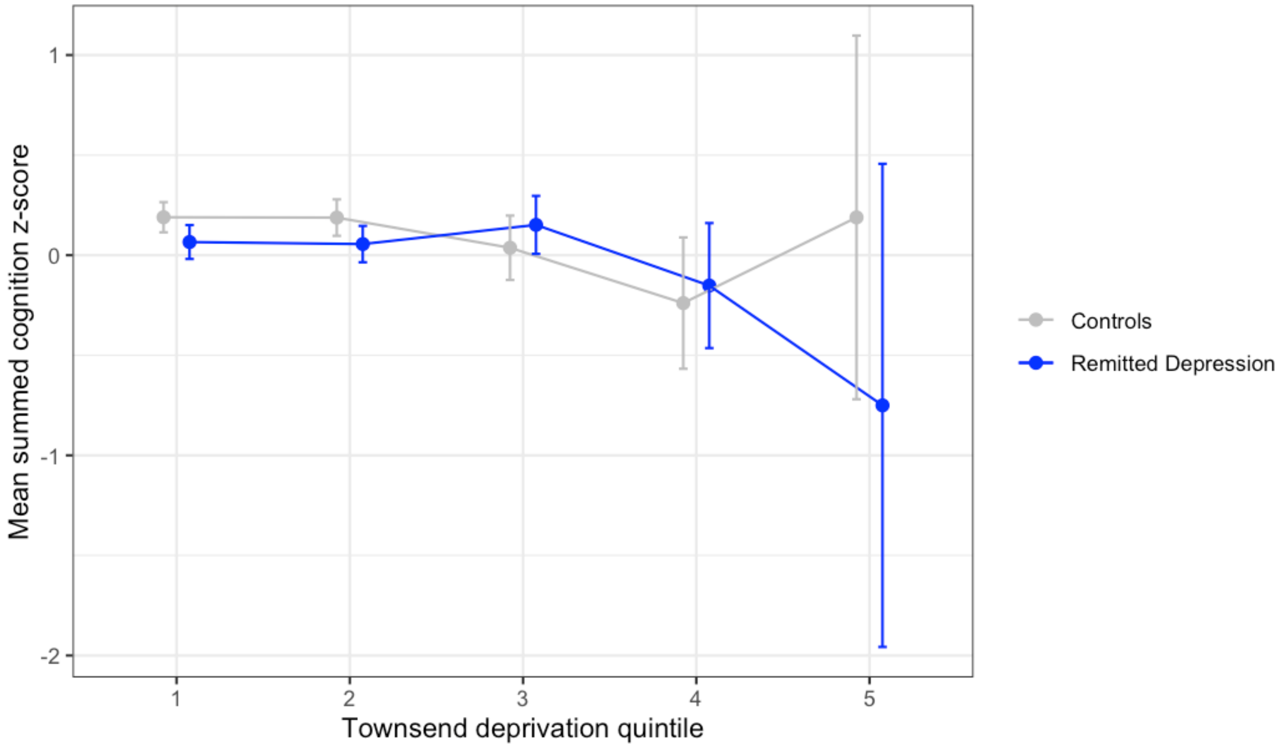

Results from Sensitivity Analyses for Primary outcome: characteristics for GP cohort and Probable Depression cohort

Primary RD (using First Occurrences and Hospital Episode Statistics (FO+HES)) and secondary RD cohorts (using GP data; and the “Probable Depression” UKB-derived algorithm data) were similar in terms of demographic, health and imaging parameters. Differences are included in Supplementary Table 2(b) and 2(c) and summarised in Supplementary Table 4. In brief, significant differences comparing RD and control participants using less stringent depressive thresholds were that (i) in the GP cohort, RD participants were more likely to be of white ethnicity than controls; (ii) in the Probable Depression cohort, RD participants were more likely to be more living in deprived postcodes and to be in the higher education group compared to controls. The Probable Depression cohort also had fewer RD participants who were in receipt of antidepressants at the imaging visit (13.9% versus 26.2% (GP) and 34.9% (FO+HES)). However, for both the GP and Probable Depression RD cohort, compared to their control participants, there were no significant differences in performance on cognitive behavioural tasks (baseline summed z scores or on the individual domain level; ps>0.05) (see Supplementary Table 5(a)). This contrasts to that seen in the primary FO+HES RD group, where compared to controls, RD participants had a lower overall performance on cognitive tasks compared to controls.

Results from Sensitivity Analyses for Primary outcome: (i) Summed cognitive score impact on future risk of depression for GP cohort:

N=1039 in RD cohort + 1039 in matched controls = 2078.

372 (35.8%) in RD group and 177 (17%) in control group developed depression longitudinally after imaging visit

Incomplete cognitive data for 808 across both groups (406 in controls and 402 in RD cohort)

The composite cognition measure did not significantly explain group-level differences in the risk of future depression (z=-0.45, p=0.64), and there was no significant interaction between composite cognition score and group (z=-0.61, p=0.54). Models without the inclusion of group identified that there was no general association between composite cognition score and future depression across the two groups (p=0.91).

Results from Sensitivity Analyses for Primary outcome: (ii) Summed cognitive score results for Probable Depression cohort:

N=2252 in RD cohort + 2252 in matched controls = 4504.

616 (27%) in RD group and 290 (13%) in control group developed depression longitudinally after imaging visit

Incomplete cognitive data for 1724 across both groups (883 in controls and 841 in RD cohort)

The composite cognition measure did not significantly explain group-level differences in the risk of future depression (z=0.54, p=0.58), and there was no significant interaction between composite cognition score and group (z=0.64, p=0.52). Models without the inclusion of group identified that there was no general association between composite cognition score and future depression across the two groups (p=0.11).

Supplementary Figure 7: Future depression risk relating to composite cognition scores at imaging: (A) using GP records to define past depression; (B) using the Probable Depression algorithm to define past depression

A

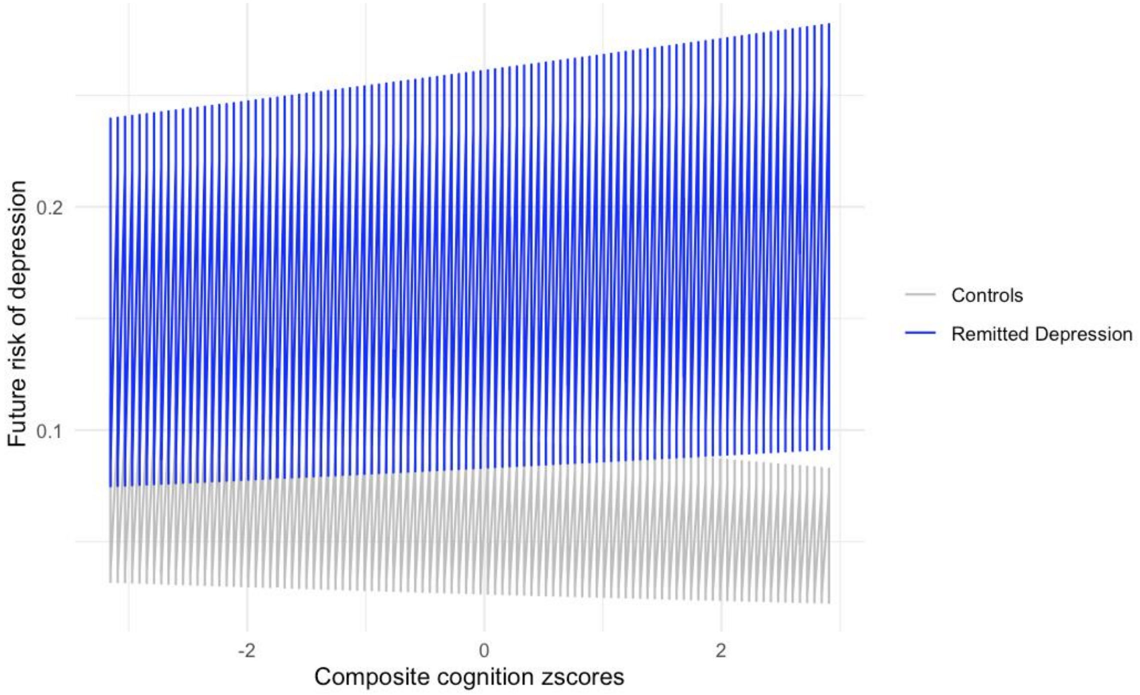

B

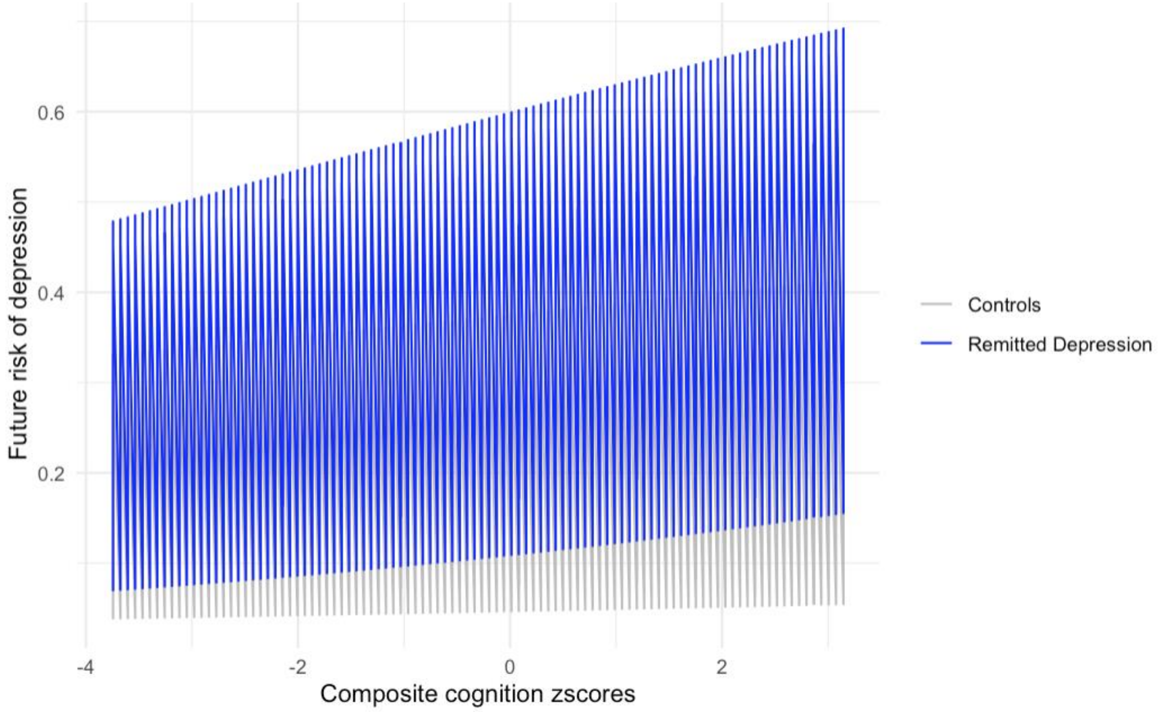

Future depression expressed as risk </=1. Cognitive scores expressed as a z score. Confidence intervals around the mean risk shown. (A) composite score across all dimensions; (B) memory scores (based on paired associate learning scores) (B) executive functioning scores (based on digit span, TMT B-A, tower arranging scores); (C) reasoning scores (based on fluid intelligence, matrix pattern puzzles scores); (D) processing speed scores (based on reaction time, symbol digit substitution scores)

Supplementary Figure 8: Dimensional comparison across the primary and secondary cohorts

(A) Memory

(i) FO+HES (N=3724)

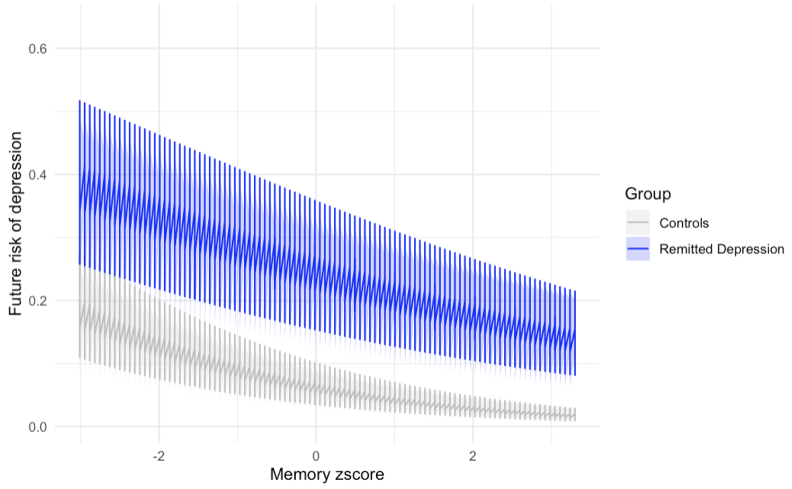

(ii) GP (N=2078)

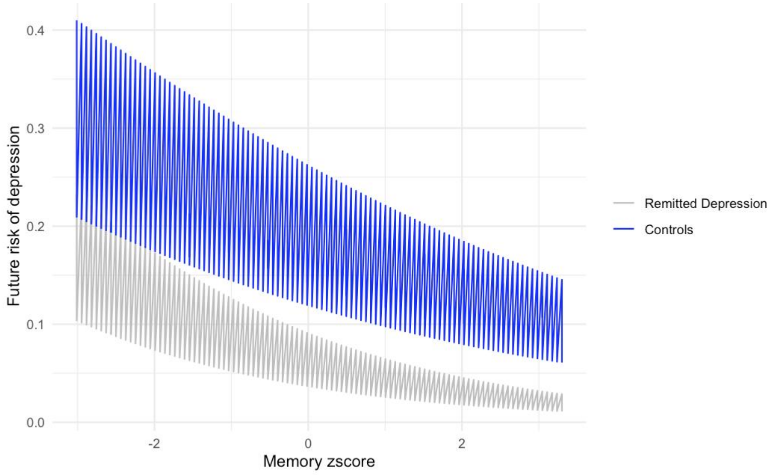

(iii) Probable Depression (N=4504)

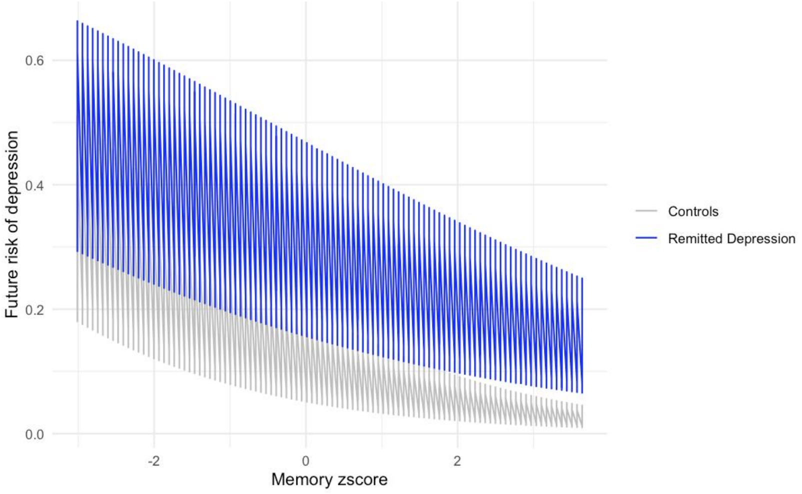

(B) Executive function

(i) FO+HES (N=2310)

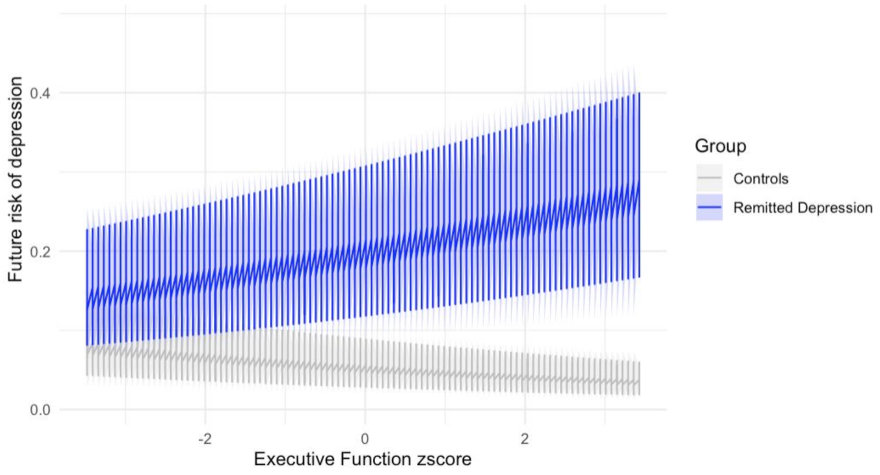

(ii) GP (N=1277)

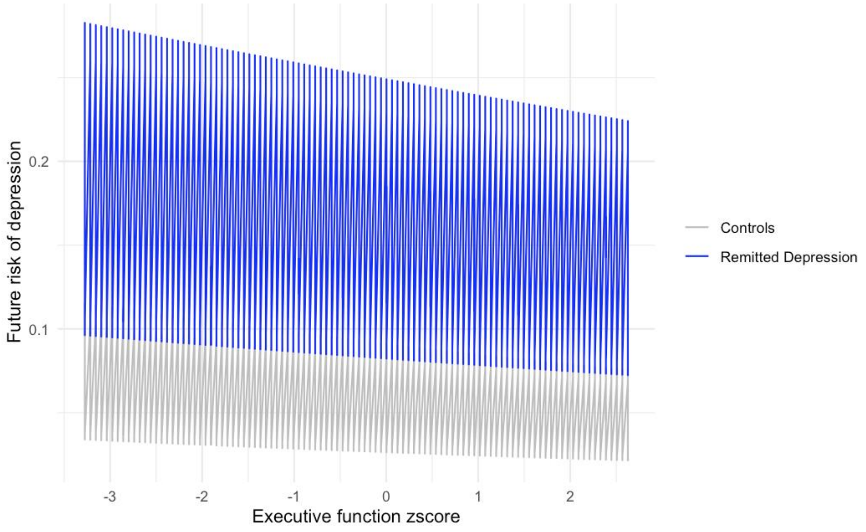

(iii) Probable Depression (N=2310)

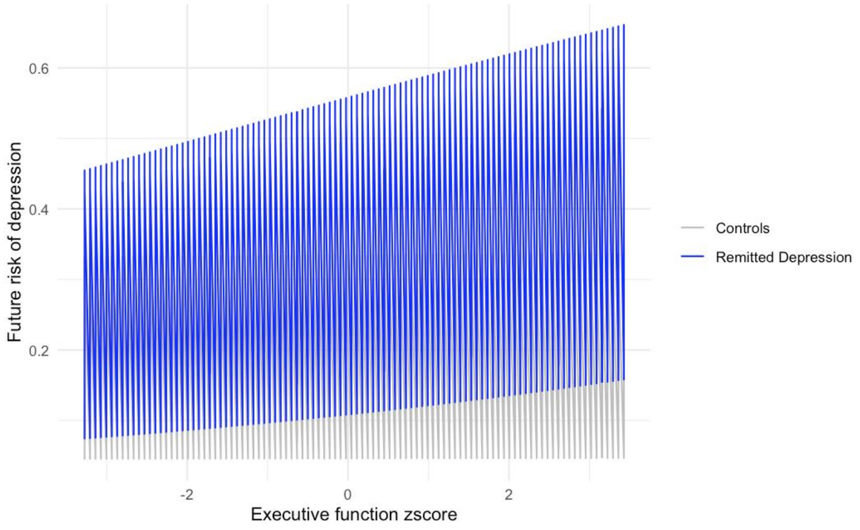

(C) Reasoning

(i) FO+HES (N=2617)

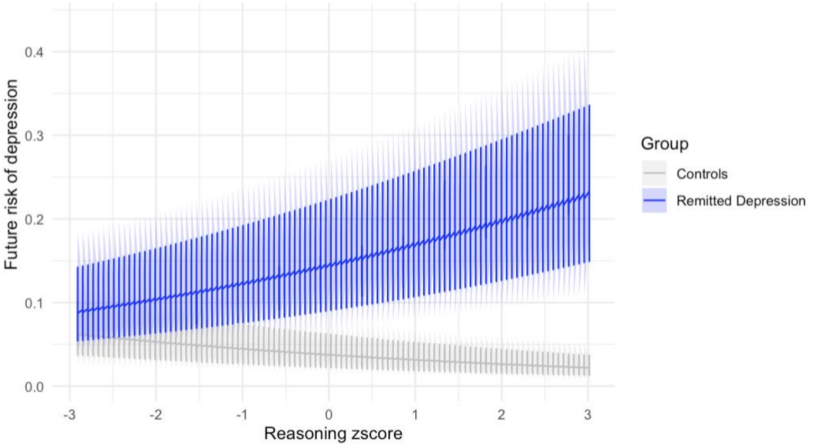

(ii) GP (N=1401)

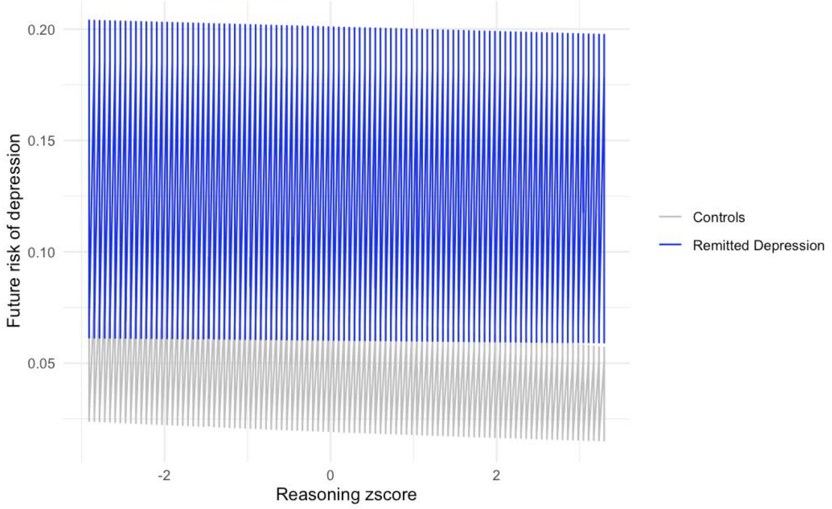

(iii) Probable Depression (N=2617)

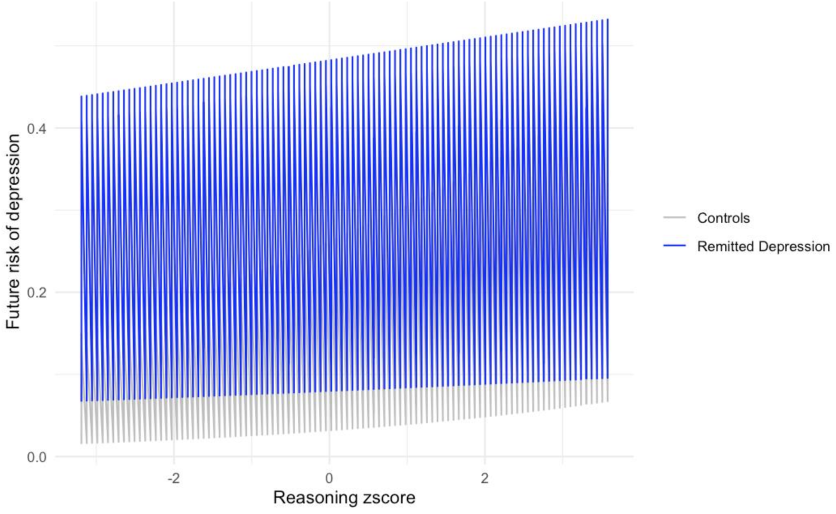

(D) Processing speed

(i) FO+HES (N=2639)

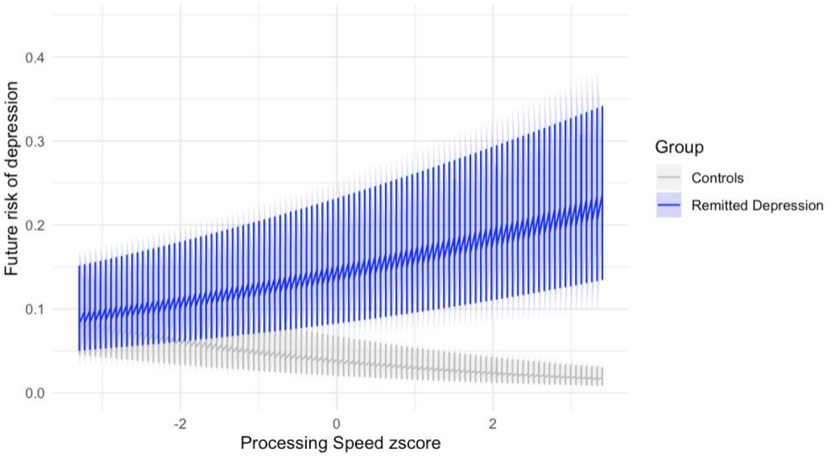

(ii) GP (N=1422)

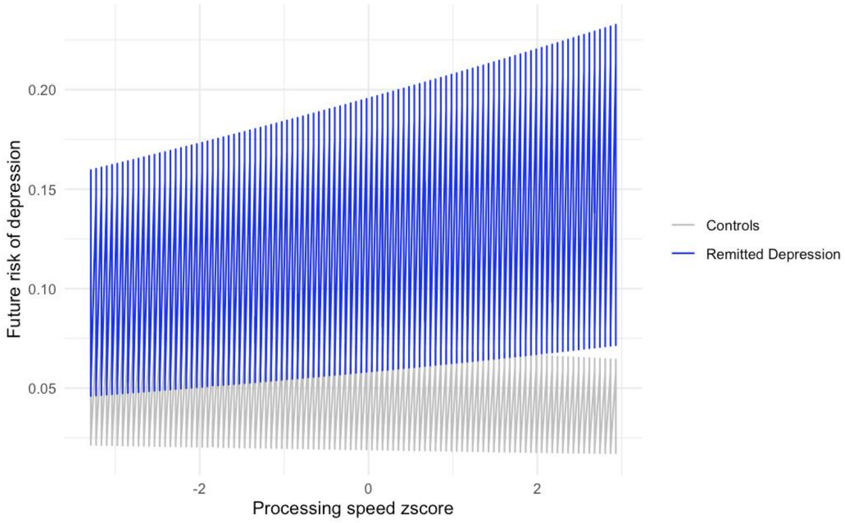

(iii) Probable Depression (N=2639)

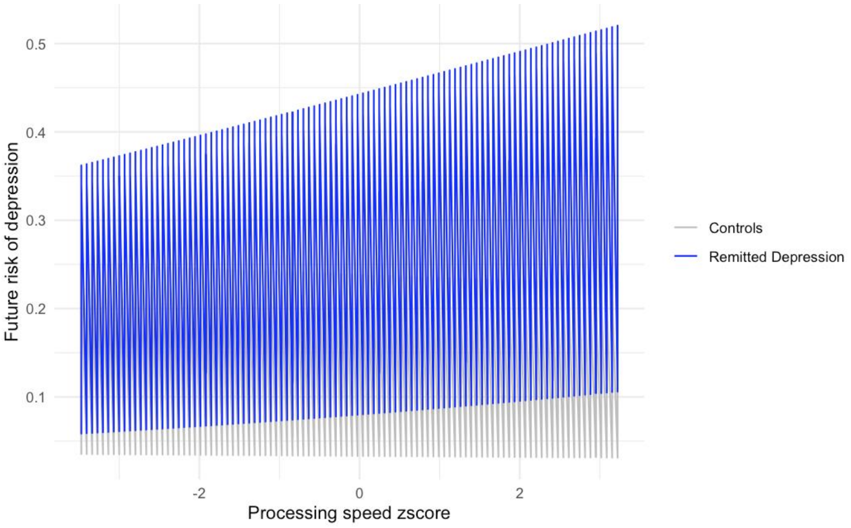

Robustness check 1) Effect on antidepressant use on cognition at baseline / imaging within remitted depression group

Supplementary Table 10: Proportion of remitted depression cohort using antidepressants

No use	Baseline only	Baseline & imaging	Imaging only
52.52%	12.62%	14.45%	20.41%

Antidepressant use at baseline and imaging did not appear to have a significant effect on summed cognitive scores at imaging:

i) Comparing no use / antidepressants at baseline only / antidepressants at baseline and imaging / antidepressants only at imaging; p=0.551

ii) Comparing antidepressants at baseline and imaging versus antidepressants at baseline only (i.e.persistence vs non-persistence); p=0.23

Of those who relapsed, 231 (12% of total RD cohort; 37% of RD cohort who relapsed) were using antidepressants.

We did not investigate a group-effect of antidepressant use as this was outside the scope of our analyses and not pre-specified.

Robustness check 2) Overlap between cohorts

Supplementary Table 11: Overlap between primary and secondary cohorts

(i) **FO+HES and GP cohort**

In both subsets	482
Only in FO+HES	1380
Only in GP	557

(ii) **FO+HES and Probable Depression cohort**

In both subsets	360
Only in FO+HES	1502
Only in Probable Depression	1892

(iii) **GP and Probable Depression cohort**

In both subsets	242
Only in GP	557
Only in Probable Depression	2010

Robustness check 3) Relapse rate across different confounders

Out of 1862 participants who fulfilled criteria as defined above for the primary RD cohort (and had full data for control variables), 623 (33%) had a relapse of depression following the UK Biobank visit where cognitive and imaging data were collected. For 1862 matched controls, 240 (13%) had a new episode of depression at the equivalent time point.

Of 863 individuals who had an episode of depression after imaging, we can break this down into how this is affected across different non-continuous confounders (%):

Supplementary Table 12: Demographics of participants in primary cohort who developed depression after imaging (RD cohort = 623; control cohort = 240)

(i) Ethnicity

	White	Black	Mixed / other
Controls	98.3	0.8	0.8
RD cohort	98.3	0.1	1.4

(ii) Education

	Uni	A levels	O levels	CSEs	NVQ/HND	Other professional	None of the above
Controls	47.5	14.5	17.0	4.5	5.0	5.0	6.3
RD cohort	49.5	14.4	18.9	4.0	4.7	5.3	3.0

(iii) Deprivation

	Least				Most
Controls	47.9	37.5	12.5	1.7	0.4
RD cohort	44.6	37.5	13.0	4.7	0.2

(iv) Smoking at imaging

	Non-smoker	Smoker
Controls	62.9	37.1
RD cohort	57.1	42.9

(v) Epilepsy at imaging

	No	Yes
Controls	99.6	0.4
RD cohort	98.7	1.3

(vi) Chronic IHD at imaging

	No	Yes
Controls	97.9	2.1
RD cohort	94.5	5.5

(vii) Cerebrovascular disease at imaging

	No	Yes
Controls	100	0
RD cohort	97.8	2.2

(viii) Cancer before imaging

	No	Yes
Controls	92.2	8.8
RD cohort	85.4	14.6

Supplementary Table 13(a): Comparison between remitted depressed cohort with complete data for control variables (n=1039) and matched control cohort (n=1039) for GP cohort

Characteristic / health factor	Estimate	P value
Ethnicity	7.35 (chi-sq)	**0.03**
Education	5.50 (chi-sq)	0.48
Deprivation (Townsend Index)	8.77 (chi-sq)	0.07
BMI	26.6 (F)	**<0.001**
Smoking	17.9 (chi-sq)	**<0.001**
Epilepsy	0.57 (chi-sq)	0.45
Cerebrovascular disease	5.05 (chi-sq)	0.02
Ischaemic heart disease	7.12 (chi-sq)	**0.008**
Previous cancer	3.04 (chi-sq)	0.08
Alcohol	0.21 (F)	0.65

Supplementary Table 13(b): Comparison between remitted depressed cohort with complete data for control variables (n=2252) and matched control cohort (n=2252) for Probable Depression cohort

Characteristic / health factor	Estimate	P value
Ethnicity	1.01 (chi-sq)	0.60
Education	18.3 (chi-sq)	0.005
Deprivation (Townsend Index)	13.4 (chi-sq)	**0.01**
BMI	32.3 (F)	**<0.001**
Smoking	24.1 (chi-sq)	**<0.001**
Epilepsy	1.89 (chi-sq)	0.17
Cerebrovascular disease	1.53 (chi-sq)	0.17
Ischaemic heart disease	0.78 (chi-sq)	0.37

Previous cancer	6.23 (chi-sq)	0.01
Alcohol	0.15 (F)	0.70

Supplementary Table 13(c): Differences between RD and matched control primary cohorts when compared with other secondary RD and matched control cohorts

	Estimate (p value)			***Direction if significant (and if similar across cohorts)***
**Baseline measure**	*FO+HES cohort (n=1862)*	*GP cohort (n=1039)*	*Prob Dep cohort (n=2252)*	
Ethnicity (x-sq)	8.40 (p=0.02)	7.35 (p=0.03)	1.01 (p=0.60)	More likely to be white in RD cohort than controls (FO+HES and GP significant)
Townsend (x-sq)	11.4 (p=0.02)	5.50 (p=0.48)	13.4 (p=0.01)	Least deprived (TDI 1) higher proportion in controls (FO+HES and Prob Dep significant)
Education (x-sq)	5.49 (p=0.51)	5.88 (p=0.44)	18.3 (p=0.005)	Higher proportion of higher education groups in depressed group (Prob Dep significant)
White matter (F)	0.01 (p=0.91)	6.15 (p=0.01)	0.66 (p=0.41)	WM volume tending to be reduced in RD cohorts (GP significant)
Grey matter (F)	7.64 (p=0.005)	1.19(p=0.27)	0.15 (p=0.70)	GM volume tending to be reduced in RD cohorts (FO+HES significant)
Antidepressants (% in RD cohort)	34.9	26.2	13.9	Lower proportion on antidepressants in Prob Dep cohort

Supplementary Table 13(d): Differences with other cohorts in terms of cognitive functioning at the imaging visit

(i) GP cohort versus matched controls

Group	Statistic	summed_zscores	processing_speed	reasoning	executive_function	memory
<dbl>	<chr>	<dbl>	<dbl>	<dbl>	<dbl>	<dbl>
0	Mean	0.15918491	0.22382478	0.07848174	0.10067352	−1.022562
0	Median	0.23704000	0.24913572	0.19389773	0.08780593	−1.357533
0	SD	0.97950201	0.94920220	1.02660274	0.97898818	1.062271
1	Mean	0.08286983	0.09367562	0.04824172	0.05344319	−1.043132
1	Median	0.11734564	0.18744890	0.19389773	0.08593540	−1.357533
1	SD	0.99868403	1.00112404	0.98072864	1.01083480	1.025205

(ii) Probable Depression versus matched controls

Group	Statistic	summed_zscores	processing_speed	reasoning	executive_function	memory
<dbl>	<chr>	<dbl>	<dbl>	<dbl>	<dbl>	<dbl>
0	Mean	0.1080188	0.11321992	0.1170804	0.06566302	−1.081462
0	Median	0.1775595	0.15520730	0.1938977	0.10482261	−1.357533
0	SD	0.9370549	0.93820154	0.9627869	0.96827770	1.017235
1	Mean	0.1286556	0.07979041	0.1823660	0.09522636	−1.073180
1	Median	0.1389008	0.10280593	0.1938977	0.08593540	−1.357533
1	SD	0.9322750	0.96041311	0.9456086	0.97686839	1.024716

Supplementary Table 14: Imaging parameters at baseline (n=1554)

**Parameter**	**Matched control cohort (1554)**	**Primary RD cohort (1554)**	**Estimate**	**P value**
Head motion	0.115 (0.049)	0.129 (0.064)	47.4 (F)	**<0.001**
Total brain volume	1502067 (73042)	1498992 (73460)	1.37 (F)	0.24
White matter volume	701924 (40096)	703397 (39864)	1.05 (F)	0.31
Grey matter volume	800144 (47114)	795795 (48417)	7.05 (F)	**0.008**
Cortical volume	623385 (40686)	620398 (41252)	4.13 (F)	**0.042**
CSF volume	44309 (18885)	44837 (18255)	0.63 (F)	0.42

Supplementary Table 15(a): Regional structural volumes of the Triple Network for RD cohort with full imaging data (Group 1; n=1554) and matched control cohort (Group 0; n=1554)

Variable <chr>	Group <fctr>	Median <dbl>	Mean <dbl>	SD <dbl>
Left_ACC	0	4994.405	5124.508	1035.9283
Left_ACC	1	4967.240	5108.374	1051.2684
Left_PCC	0	5095.245	5182.638	793.1059
Left_PCC	1	5102.965	5167.000	753.5814
Left_amygdala	0	1832.235	1845.174	240.1031
Left_amygdala	1	1812.420	1823.077	253.1788
Left_angular_gyrus	0	4043.980	4118.415	772.9186
Left_angular_gyrus	1	3999.140	4063.848	799.3591
Left_hippocampus	0	4196.240	4213.731	416.0895
Left_hippocampus	1	4164.205	4188.068	437.1669
Left_middle_frontal_gyrus	0	9883.330	10067.675	1817.2247
Left_middle_frontal_gyrus	1	9923.750	10062.068	1815.2390
Left_precuneous_cortex	0	10033.000	10092.352	1453.8796
Left_precuneous_cortex	1	10010.500	10114.786	1426.6253
Left_superior_frontal_gyrus	0	1866.090	1889.529	340.7915
Left_superior_frontal_gyrus	1	1844.045	1866.540	332.0520
Left_supramarginal_gyrus_ant	0	3119.220	3176.185	659.7727
Left_supramarginal_gyrus_ant	1	3142.425	3183.994	679.0305
Right_ACC	0	5495.655	5631.786	1134.6358
Right_ACC	1	5473.895	5634.504	1144.6218
Right_PCC	0	5341.395	5414.866	803.3718
Right_PCC	1	5327.005	5388.687	754.0162
Right_amygdala	0	2105.360	2116.700	269.3252
Right_amygdala	1	2087.835	2096.361	278.0129
Right_angular_gyrus	0	5417.985	5523.250	1094.5016
Right_angular_gyrus	1	5452.500	5510.461	1131.0442
Right_hippocampus	0	4367.465	4377.533	428.7142
Right_hippocampus	1	4332.720	4357.555	445.5454
Right_middle_frontal_gyrus	0	9473.960	9565.394	1715.7013
Right_middle_frontal_gyrus	1	9437.950	9518.026	1700.6931
Right_precuneous_cortex	0	10477.100	10543.501	1542.0331
Right_precuneous_cortex	1	10517.800	10573.605	1550.0215
Right_superior_frontal_gyrus	0	1875.255	1900.396	336.9625
Right_superior_frontal_gyrus	1	1842.825	1874.929	329.1647
Right_supramarginal_gyrus_ant	0	4634.575	4661.477	848.9569
Right_supramarginal_gyrus_ant	1	4589.725	4644.534	911.3952

Supplementary Table 15(b): Group differences in structural imaging for RD cohort with full imaging data (n=1554) and matched control cohort (n=1554)

Controlled for total brain volume and age and sex orthogonally as well as remainder of pre-specified covariates, and also shown with adjustment for FDR across 16 tests

	Variable<br><chr>	t_value<br><dbl>	p_value<br><dbl>	p_adjusted<br><dbl>
t	Left_ACC	0.43092855	0.66655023	0.8515383
t1	Left_middle_frontal_gyrus	0.08605197	0.93143065	0.9314306
t2	Left_precuneous_cortex	−0.43416804	0.66419659	0.8515383
t3	Left_amygdala	2.49642479	0.01259681	0.1007745
t4	Left_PCC	0.56347461	0.57315252	0.8515383
t5	Left_supramarginal_gyrus_ant	−0.32514140	0.74509597	0.8515383
t6	Left_hippocampus	1.67624636	0.09379083	0.2501089
t7	Left_superior_frontal_gyrus	1.90463512	0.05691987	0.2276795

	Variable<br><chr>	t_value<br><dbl>	p_value<br><dbl>	p_adjusted<br><dbl>
t	Right_ACC	−0.06647656	0.94700270	0.9470027
t1	Right_middle_frontal_gyrus	0.77295735	0.43960644	0.6763622
t2	Right_precuneous_cortex	−0.54277177	0.58732590	0.6763622
t3	Right_amygdala	2.07136862	0.03840683	0.1536273
t4	Right_PCC	0.93665687	0.34900820	0.6763622
t5	Right_supramarginal_gyrus_ant	0.53626079	0.59181692	0.6763622
t6	Right_hippocampus	1.27372700	0.20285563	0.5409483
t7	Right_superior_frontal_gyrus	2.13129507	0.03314318	0.1536273

Supplementary Table 16: Composite cognition scores and association with cross-sectional brain regional volumes across all participants (n=1554)

Controlled for total brain volume and age and sex orthogonally as well as remainder of pre-specified covariates, and also shown with adjustment for FDR across 16 tests

Brain region (volume)	t value	Main effect of region (p value, FDR corrected across 16 tests)	Interaction (p value, FDR corrected across 16 tests)
ACC(L)	-0.04	0.97	0.92
ACC(R)	0.12	0.97	0.92
Amygdala (L)	4.63	**<0.001**	0.92
Amygdala (R)	5.01	**<0.001**	0.92
Hippocampus (L)	3.34	**0.005**	0.92
Hippocampus (R)	2.77	**0.02**	0.92
Middle frontal gyrus (L)	2.28	**0.049**	0.96
Middle frontal gyrus (R)	2.25	**0.049**	0.92
Precuneous (L)	2.58	**0.03**	0.93
Precuneous (R)	1.91	0.09	0.96
PCC(L)	2.44	**0.039**	0.96
PCC(R)	2.14	0.058	0.96
Supramarginal gyrus (L)	0.05	0.97	0.92
Supramarginal gyrus (R)	1.00	0.39	0.92
Superior frontal gyrus (L)	1.22	0.33	0.92
Superior frontal gyrus (R)	1.02	0.39	0.92

Supplementary Table 17: Impact of brain regional volumes within Triple Network on future risk of depression for RD (n=1554) and matched control cohorts (n=1554)

Controlled for total brain volume and age and sex orthogonally as well as remainder of pre-specified covariates, and also shown with adjustment for FDR across 16 tests

(i) Main effect of group

Variable <chr>	Original_p_value <dbl>	Adjusted_p_value <dbl>
Right_ACC	0.41006514	0.5467535
Left_ACC	0.26619212	0.4937383
Right_Amygdala	0.29638835	0.4937383
Left_Amygdala	0.16514579	0.4937383
Right_Hippocampus	0.98484590	0.9848459
Left_Hippocampus	0.31293719	0.4937383
Right_MFG	0.09848766	0.4937383
Left_MFG	0.44892668	0.5525251
Right_Precuneous	0.65701875	0.7508786
Left_Precuneous	0.32114323	0.4937383
Right_PCC	0.16261193	0.4937383
Left_PCC	0.33839603	0.4937383
Right_SMG	0.33944509	0.4937383
Left_SMG	0.98404628	0.9848459
Right_SFG	0.24371991	0.4937383
Left_SFG	0.21706270	0.4937383

(ii) Group*regional volume interaction

Variable <chr>	Original_p_value <dbl>	Adjusted_p_value <dbl>
Right_ACC_Group	0.48749126	0.9230541
Left_ACC_Group	0.55556075	0.9230541
Right_Amygdala_Group	0.28951204	0.9230541
Left_Amygdala_Group	0.36938795	0.9230541
Right_Hippocampus_Group	0.84246892	0.9230541
Left_Hippocampus_Group	0.63834350	0.9230541
Right_MFG_Group	0.04065445	0.6504711
Left_MFG_Group	0.27408674	0.9230541
Right_Precuneous_Group	0.88667006	0.9230541
Left_Precuneous_Group	0.75543453	0.9230541
Right_PCC_Group	0.44276723	0.9230541
Left_PCC_Group	0.67216935	0.9230541
Right_SMG_Group	0.59916201	0.9230541
Left_SMG_Group	0.92305413	0.9230541
Right_SFG_Group	0.79064196	0.9230541
Left_SFG_Group	0.33317918	0.9230541

Supplementary Table 18(a): Impact of rsfMRI functional connectivity (nodes) on cognitive scores within Triple Network

Controlled for total brain volume and age and sex orthogonally as well as remainder of pre-specified covariates, and also shown with adjustment for FDR across 11 tests

Node	Description	Effect	Estimate	P_value	P_value_FDR
X1	X1 (DMN)	Interaction	-0.0181	0.8517	0.9925
X1	X1 (DMN)	Main effect	-0.0125	0.8497	0.9868
X3	X3 (SN)	Interaction	-0.0025	0.9826	0.9925
X3	X3 (SN)	Main effect	0.0637	0.4153	0.9136
X5	X5 (cEN / DMN)	Interaction	-0.2411	0.0234	0.1287
X5	X5 (cEN / DMN)	Main effect	0.1602	0.0304	0.3340
X6	X6 (cEN / DMN)	Interaction	-0.0865	0.4590	0.9871
X6	X6 (cEN / DMN)	Main effect	-0.0013	0.9868	0.9868
X7	X7 (DMN / cEN)	Interaction	0.1874	0.0866	0.3177
X7	X7 (DMN / cEN)	Main effect	-0.0997	0.1650	0.6050
X9	X9 (DMN / cEN)	Interaction	-0.0343	0.6886	0.9925
X9	X9 (DMN / cEN)	Main effect	-0.0141	0.8178	0.9868
X13	X13 (SN / DMN)	Interaction	-0.0355	0.7832	0.9925
X13	X13 (SN / DMN)	Main effect	-0.0111	0.9019	0.9868
X14	X14 (DMN / cEN)	Interaction	-0.0977	0.5384	0.9871
X14	X14 (DMN / cEN)	Main effect	0.1019	0.3575	0.9136
X16	X16 (cEN)	Interaction	0.2619	0.0187	0.1287
X16	X16 (cEN)	Main effect	-0.1308	0.0886	0.4872
X20	X20 (DMN / cEN)	Interaction	-0.0011	0.9925	0.9925
X20	X20 (DMN / cEN)	Main effect	-0.0281	0.7149	0.9868
X21	X21 (cEN / DMN)	Interaction	0.1161	0.3642	0.9871
X21	X21 (cEN / DMN)	Main effect	-0.0022	0.9810	0.9868

Supplementary Table 18(b): Impact of rsfMRI functional connectivity (edges) on cognitive scores within Triple Network

Controlled for total brain volume and age and sex orthogonally as well as remainder of pre-specified covariates, and also shown with adjustment for FDR across 54 tests

Edge	Description	Effect	Estimate	P_value	P_value_FDR
V2	V2 (nodes 1-3) DMN – SN	Interaction	-0.0723	0.0930	0.7846
V2	V2 (nodes 1-3) DMN – SN	Main effect	0.0410	0.1756	0.5267
V7	V7 (nodes 1-5) DMN – cEN / DMN	Interaction	0.0153	0.7300	0.9987
V7	V7 (nodes 1-5) DMN – cEN / DMN	Main effect	-0.0139	0.6380	0.8402
V9	V9 (nodes 3-5) SN – cEN / DMN	Interaction	-0.0344	0.4130	0.8174
V9	V9 (nodes 3-5) SN – cEN / DMN	Main effect	0.0034	0.9043	0.9386
V11	V11 (nodes 1-6) DMN – cEN / DMN	Interaction	0.0486	0.3245	0.8174
V11	V11 (nodes 1-6) DMN – cEN / DMN	Main effect	-0.0086	0.8079	0.9363
V13	V13 (nodes 3-6) SN – cEN / DMN	Interaction	0.0663	0.1711	0.8048
V13	V13 (nodes 3-6) SN – cEN / DMN	Main effect	-0.0318	0.3439	0.7020
V15	V15 (nodes 5-6) cEN / DMN – cEN / DMN	Interaction	0.0677	0.1017	0.7846
V15	V15 (nodes 5-6) cEN / DMN – cEN / DMN	Main effect	-0.0409	0.1592	0.5057
V16	V16 (nodes 1-7) DMN – DMN / cEN	Interaction	0.0030	0.9474	0.9987
V16	V16 (nodes 1-7) DMN – DMN / cEN	Main effect	0.0038	0.9025	0.9386
V18	V18 (nodes 3-7) SN – DMN / cEN	Interaction	0.0043	0.9234	0.9987
V18	V18 (nodes 3-7) SN – DMN / cEN	Main effect	-0.0219	0.4473	0.7142
V20	V20 (nodes 5-7) cEN / DMN – DMN / cEN	Interaction	-0.0479	0.2735	0.8048
V20	V20 (nodes 5-7) cEN / DMN – DMN / cEN	Main effect	0.0547	0.0713	0.3775
V21	V21 (nodes 6-7) cEN / DMN – DMN / cEN	Interaction	0.0516	0.2832	0.8048
V21	V21 (nodes 6-7) cEN / DMN – DMN / cEN	Main effect	-0.0317	0.3558	0.7020
V29	V29 (nodes 1-9) DMN – DMN / cEN	Interaction	0.0379	0.4019	0.8174
V29	V29 (nodes 1-9) DMN – DMN / cEN	Main effect	-0.0357	0.2584	0.6296
V31	V31 (nodes 3-9) SN – DMN / cEN	Interaction	-0.0140	0.7751	0.9987
V31	V31 (nodes 3-9) SN – DMN / cEN	Main effect	0.0217	0.5220	0.7618
V33	V33 (nodes 5-9) cEN / DMN – DMN / cEN	Interaction	-0.0016	0.9713	0.9987
V33	V33 (nodes 5-9) cEN / DMN – DMN / cEN	Main effect	-0.0159	0.6104	0.8240
V34	V34 (nodes 6-9) cEN / DMN – DMN / cEN	Interaction	-0.0287	0.5393	0.9395
V34	V34 (nodes 6-9) cEN / DMN – DMN / cEN	Main effect	0.0254	0.4312	0.7142
V35	V35 (nodes 7-9) DMN / cEN – cEN	Interaction	-0.0424	0.3555	0.8174
V35	V35 (nodes 7-9) DMN / cEN – cEN	Main effect	0.0578	0.0726	0.3775
V67	V67 (nodes 1-13) DMN – SN / DMN	Interaction	-0.0435	0.4238	0.8174
V67	V67 (nodes 1-13) DMN – SN / DMN	Main effect	0.0088	0.8149	0.9363
V69	V69 (nodes 3-13) SN – DMN	Interaction	-0.0001	0.9987	0.9987
V69	V69 (nodes 3-13) SN – DMN	Main effect	-0.0652	0.0839	0.3775
V71	V71 (nodes 5-13) cEN / DMN – DMN	Interaction	0.0845	0.0573	0.7846
V71	V71 (nodes 5-13) cEN / DMN – DMN	Main effect	-0.0560	0.0646	0.3775
V72	V72 (nodes 6-13) cEN / DMN – DMN	Interaction	0.0114	0.8151	0.9987
V72	V72 (nodes 6-13) cEN / DMN – DMN	Main effect	-0.0483	0.1504	0.5057

Edge	Description	Effect	Estimate	P_value	P_value_FDR
V73	V73 (nodes 7-13) DMN / cEN – DMN	Interaction	-0.0978	0.0647	0.7846
V73	V73 (nodes 7-13) DMN / cEN – DMN	Main effect	0.0450	0.2313	0.5948
V75	V75 (nodes 9-13) cEN – DMN	Interaction	-0.0711	0.1769	0.8048
V75	V75 (nodes 9-13) cEN – DMN	Main effect	0.0206	0.5625	0.7980
V79	V79 (nodes 1-14) DMN – DMN / cEN	Interaction	0.0065	0.8850	0.9987
V79	V79 (nodes 1-14) DMN – DMN / cEN	Main effect	0.0280	0.3640	0.7020
V81	V81 (nodes 3-14) SN – DMN / cEN	Interaction	0.0750	0.2097	0.8048
V81	V81 (nodes 3-14) SN – DMN / cEN	Main effect	-0.0173	0.6755	0.8569
V83	V83 (nodes 5-14) cEN / DMN – DMN / cEN	Interaction	0.0618	0.2671	0.8048
V83	V83 (nodes 5-14) cEN / DMN – DMN / cEN	Main effect	-0.0044	0.9105	0.9386
V84	V84 (nodes 6-14) cEN / DMN – DMN / cEN	Interaction	-0.0028	0.9615	0.9987
V84	V84 (nodes 6-14) cEN / DMN – DMN / cEN	Main effect	0.0022	0.9558	0.9558
V85	V85 (nodes 7-14) DMN / cEN – DMN / cEN	Interaction	-0.0007	0.9893	0.9987
V85	V85 (nodes 7-14) DMN / cEN – DMN / cEN	Main effect	0.0681	0.0654	0.3775
V87	V87 (nodes 9-14) cEN – DMN / cEN	Interaction	-0.0442	0.4430	0.8249
V87	V87 (nodes 9-14) cEN – DMN / cEN	Main effect	0.0486	0.2179	0.5948
V91	V91 (nodes 13-14) DMN – DMN / cEN	Interaction	-0.0525	0.3802	0.8174
V91	V91 (nodes 13-14) DMN – DMN / cEN	Main effect	0.0272	0.5056	0.7585
V106	V106 (nodes 1-16) DMN – cEN	Interaction	-0.0399	0.3204	0.8174
V106	V106 (nodes 1-16) DMN – cEN	Main effect	0.0274	0.3241	0.7001
V108	V108 (nodes 3-16) SN – cEN	Interaction	0.0122	0.7906	0.9987
V108	V108 (nodes 3-16) SN – cEN	Main effect	-0.0238	0.4497	0.7142
V110	V110 (nodes 5-16) cEN / DMN – cEN	Interaction	-0.0046	0.9145	0.9987
V110	V110 (nodes 5-16) cEN / DMN – cEN	Main effect	-0.0217	0.4657	0.7186
V111	V111 (nodes 6-16) cEN / DMN – cEN	Interaction	-0.0705	0.0892	0.7846
V111	V111 (nodes 6-16) cEN / DMN – cEN	Main effect	0.0618	0.0318	0.3775
V112	V112 (nodes 7-16) DMN / cEN – cEN	Interaction	-0.0031	0.9395	0.9987
V112	V112 (nodes 7-16) DMN / cEN – cEN	Main effect	-0.0060	0.8344	0.9386
V114	V114 (nodes 9-16) cEN – cEN	Interaction	-0.0541	0.2394	0.8048
V114	V114 (nodes 9-16) cEN – cEN	Main effect	0.0696	0.0265	0.3775
V118	V118 (nodes 13-16) DMN – cEN	Interaction	-0.0835	0.1278	0.8048
V118	V118 (nodes 13-16) DMN – cEN	Main effect	0.0068	0.8601	0.9386
V119	V119 (nodes 14 – 16) cEN – cEN	Interaction	0.0179	0.7218	0.9987
V119	V119 (nodes 14 – 16) cEN – cEN	Main effect	0.0286	0.4177	0.7142
V172	V172 (nodes 1-20) DMN – DMN / cEN	Interaction	-0.0200	0.6531	0.9987
V172	V172 (nodes 1-20) DMN – DMN / cEN	Main effect	-0.0117	0.7071	0.8608

Edge	Description	Effect	Estimate	P_value	P_value_FDR
V174	V174 (nodes 3-20) SN – DMN / CEN	Interaction	-0.0291	0.5632	0.9505
V174	V174 (nodes 3-20) SN – DMN / CEN	Main effect	-0.0274	0.4344	0.7142
V176	V176 (nodes 5-20) cEN / DMN – DMN / cEN	Interaction	-0.0529	0.2480	0.8048
V176	V176 (nodes 5-20) cEN / DMN – DMN / cEN	Main effect	0.0479	0.1350	0.4861
V177	V177 (nodes 6-20) cEN – DMN / cEN	Interaction	0.0390	0.3796	0.8174
V177	V177 (nodes 6-20) cEN – DMN / cEN	Main effect	-0.0128	0.6823	0.8569
V178	V178 (nodes 7-20) DMN / cEN – DMN / cEN	Interaction	-0.0223	0.6011	0.9836
V178	V178 (nodes 7-20) DMN / cEN – DMN / cEN	Main effect	0.0313	0.2825	0.6356
V180	V180 (nodes 9-20) cEN – DMN / cEN	Interaction	-0.0526	0.2718	0.8048
V180	V180 (nodes 9-20) cEN – DMN / cEN	Main effect	0.0609	0.0756	0.3775
V184	V184 (nodes 13-20) DMN – DMN / cEN	Interaction	-0.0046	0.9359	0.9987
V184	V184 (nodes 13-20) DMN – DMN / cEN	Main effect	0.0446	0.2682	0.6296
V185	V185 (nodes 14-20) cEN – DMN / cEN	Interaction	0.0382	0.4815	0.8667
V185	V185 (nodes 14-20) cEN – DMN / cEN	Main effect	0.0137	0.7173	0.8608
V191	V191 (nodes 1-21) DMN – cEN / DMN	Interaction	0.0564	0.2735	0.8048
V191	V191 (nodes 1-21) DMN – cEN / DMN	Main effect	-0.0813	0.0202	0.3775
V193	V193 (nodes 3-21) SN – cEN / DMN	Interaction	0.0138	0.7877	0.9987
V193	V193 (nodes 3-21) SN – cEN / DMN	Main effect	-0.0631	0.0688	0.3775
V195	V195 (nodes 5-21) cEN / DMN – cEN / DMN	Interaction	0.0540	0.2268	0.8048
V195	V195 (nodes 5-21) cEN / DMN – cEN / DMN	Main effect	-0.0541	0.0799	0.3775
V196	V196 (nodes 6-21) cEN – cEN / DMN	Interaction	0.0748	0.0484	0.7846
V196	V196 (nodes 6-21) cEN – cEN / DMN	Main effect	-0.0452	0.0950	0.3946
V197	V197 (nodes 7-21) DMN / cEN – cEN / DMN	Interaction	0.0441	0.3625	0.8174
V197	V197 (nodes 7-21) DMN / cEN – cEN / DMN	Main effect	-0.0033	0.9212	0.9386
V199	V199 (nodes 9-21) cEN – cEN / DMN	Interaction	0.0006	0.9896	0.9987
V199	V199 (nodes 9-21) cEN – cEN / DMN	Main effect	-0.0180	0.5763	0.7980
V203	V203 (nodes 13-21) DMN – cEN / DMN	Interaction	-0.0055	0.9177	0.9987
V203	V203 (nodes 13-21) DMN – cEN / DMN	Main effect	-0.0454	0.2205	0.5948
V204	V204 (nodes 14-21) cEN – cEN / DMN	Interaction	0.1037	0.0797	0.7846
V204	V204 (nodes 14-21) cEN – cEN / DMN	Main effect	-0.0685	0.1051	0.4053
V206	V206 (nodes 16-21) cEN – cEN / DMN	Interaction	0.0124	0.7938	0.9987
V206	V206 (nodes 16-21) cEN – cEN / DMN	Main effect	-0.0257	0.4309	0.7142
V210	V210 (nodes 20-21) DMN / cEN – cEN / DMN	Interaction	0.0148	0.7740	0.9987
V210	V210 (nodes 20-21) DMN / cEN – cEN / DMN	Main effect	-0.0693	0.0490	0.3775

Supplementary Table 19(a): Impact of rsfMRI functional connectivity (nodes) on future risk of depression

Controlled for total brain volume and age and sex orthogonally as well as remainder of pre-specified covariates, and also shown with adjustment for FDR across 11 tests

Node	Description	Effect	Estimate	P_value	P_value_FDR
X1	X1 (DMN)	Interaction	-0.0259	0.5262	0.9375
X1	X1 (DMN)	Main effect	0.0406	0.1637	0.7544
X3	X3 (SN)	Interaction	0.0189	0.6906	0.9375
X3	X3 (SN)	Main effect	-0.0210	0.5319	0.8359
X5	X5 (cEN / DMN)	Interaction	0.0038	0.9329	0.9375
X5	X5 (cEN / DMN)	Main effect	-0.0255	0.4200	0.7701
X6	X6 (cEN / DMN)	Interaction	0.0038	0.9375	0.9375
X6	X6 (cEN / DMN)	Main effect	-0.0029	0.9324	0.9728
X7	X7 (DMN / cEN)	Interaction	0.0583	0.2087	0.6797
X7	X7 (DMN / cEN)	Main effect	-0.0301	0.3429	0.7544
X9	X9 (DMN / cEN)	Interaction	-0.0557	0.1278	0.6797
X9	X9 (DMN / cEN)	Main effect	0.0563	0.0320	0.3520
X13	X13 (SN / DMN)	Interaction	0.0119	0.8276	0.9375
X13	X13 (SN / DMN)	Main effect	0.0057	0.8833	0.9728
X14	X14 (DMN / cEN)	Interaction	0.0076	0.9074	0.9375
X14	X14 (DMN / cEN)	Main effect	0.0485	0.3028	0.7544
X16	X16 (cEN)	Interaction	0.0539	0.2472	0.6797
X16	X16 (cEN)	Main effect	-0.0323	0.3299	0.7544
X20	X20 (DMN / cEN)	Interaction	0.0114	0.8074	0.9375
X20	X20 (DMN / cEN)	Main effect	0.0011	0.9728	0.9728
X21	X21 (cEN / DMN)	Interaction	-0.0780	0.1429	0.6797
X21	X21 (cEN / DMN)	Main effect	-0.0026	0.9472	0.9728

Supplementary Table 19(b): Impact of rsfMRI functional connectivity (edges) on future risk of depression

Controlled for total brain volume and age and sex orthogonally as well as remainder of pre-specified covariates, and also shown with adjustment for FDR across 54 tests

Edge	Description	Effect	Estimate	P_value	P_value_FDR
V2	V2 (nodes 1-3) DMN – SN	Interaction	0.0057	0.7486	0.9894
V2	V2 (nodes 1-3) DMN – SN	Main effect	0.0034	0.7921	0.9615
V7	V7 (nodes 1-5) DMN – cEN / DMN	Interaction	0.0022	0.9048	0.9894
V7	V7 (nodes 1-5) DMN – cEN / DMN	Main effect	0.0186	0.1479	0.9615
V9	V9 (nodes 3-5) SN – cEN / DMN	Interaction	-0.0137	0.4348	0.9894
V9	V9 (nodes 3-5) SN – cEN / DMN	Main effect	-0.0028	0.8192	0.9615
V11	V11 (nodes 1-6) DMN – cEN / DMN	Interaction	-0.0409	0.0474	0.9894
V11	V11 (nodes 1-6) DMN – cEN / DMN	Main effect	0.0088	0.5505	0.9615
V13	V13 (nodes 3-6) SN – cEN / DMN	Interaction	0.0015	0.9384	0.9894
V13	V13 (nodes 3-6) SN – cEN / DMN	Main effect	-0.0040	0.7788	0.9615
V15	V15 (nodes 5-6) cEN / DMN – cEN / DMN	Interaction	-0.0082	0.6426	0.9894
V15	V15 (nodes 5-6) cEN / DMN – cEN / DMN	Main effect	-0.0030	0.8081	0.9615
V16	V16 (nodes 1-7) DMN – DMN / cEN	Interaction	0.0306	0.1039	0.9894
V16	V16 (nodes 1-7) DMN – DMN / cEN	Main effect	-0.0078	0.5503	0.9615
V18	V18 (nodes 3-7) SN – DMN / cEN	Interaction	0.0111	0.5470	0.9894
V18	V18 (nodes 3-7) SN – DMN / cEN	Main effect	-0.0176	0.1629	0.9615
V20	V20 (nodes 5-7) cEN / DMN – DMN / cEN	Interaction	0.0019	0.9171	0.9894
V20	V20 (nodes 5-7) cEN / DMN – DMN / cEN	Main effect	-0.0103	0.4220	0.9615
V21	V21 (nodes 6-7) cEN / DMN – DMN / cEN	Interaction	-0.0241	0.2228	0.9894
V21	V21 (nodes 6-7) cEN / DMN – DMN / cEN	Main effect	-0.0040	0.7791	0.9615
V29	V29 (nodes 1-9) DMN – DMN / cEN	Interaction	-0.0185	0.3309	0.9894
V29	V29 (nodes 1-9) DMN – DMN / cEN	Main effect	0.0082	0.5423	0.9615
V31	V31 (nodes 3-9) SN – DMN / cEN	Interaction	0.0142	0.4828	0.9894
V31	V31 (nodes 3-9) SN – DMN / cEN	Main effect	-0.0008	0.9555	0.9842
V33	V33 (nodes 5-9) cEN / DMN – DMN / cEN	Interaction	-0.0061	0.7413	0.9894
V33	V33 (nodes 5-9) cEN / DMN – DMN / cEN	Main effect	0.0033	0.8004	0.9615
V34	V34 (nodes 6-9) cEN / DMN – DMN / cEN	Interaction	-0.0132	0.4966	0.9894
V34	V34 (nodes 6-9) cEN / DMN – DMN / cEN	Main effect	0.0191	0.1600	0.9615
V35	V35 (nodes 7-9) DMN / cEN – cEN	Interaction	-0.0007	0.9703	0.9894
V35	V35 (nodes 7-9) DMN / cEN – cEN	Main effect	-0.0060	0.6551	0.9615
V67	V67 (nodes 1-13) DMN – SN / DMN	Interaction	0.0302	0.1821	0.9894
V67	V67 (nodes 1-13) DMN – SN / DMN	Main effect	-0.0057	0.7183	0.9615
V69	V69 (nodes 3-13) SN – DMN	Interaction	-0.0113	0.6063	0.9894
V69	V69 (nodes 3-13) SN – DMN	Main effect	-0.0117	0.4590	0.9615
V71	V71 (nodes 5-13) cEN / DMN – DMN	Interaction	-0.0111	0.5478	0.9894
V71	V71 (nodes 5-13) cEN / DMN – DMN	Main effect	0.0201	0.1170	0.9615
V72	V72 (nodes 6-13) cEN / DMN – DMN	Interaction	-0.0218	0.2892	0.9894
V72	V72 (nodes 6-13) cEN / DMN – DMN	Main effect	0.0059	0.6874	0.9615

Edge	Description	Effect	Estimate	P_value	P_value_FDR
V73	V73 (nodes 7-13) DMN / cEN – DMN	Interaction	-0.0281	0.2060	0.9894
V73	V73 (nodes 7-13) DMN / cEN – DMN	Main effect	0.0076	0.6343	0.9615
V75	V75 (nodes 9-13) cEN – DMN	Interaction	-0.0077	0.7225	0.9894
V75	V75 (nodes 9-13) cEN – DMN	Main effect	0.0084	0.5791	0.9615
V79	V79 (nodes 1-14) DMN – DMN / cEN	Interaction	0.0134	0.4707	0.9894
V79	V79 (nodes 1-14) DMN – DMN / cEN	Main effect	0.0014	0.9141	0.9783
V81	V81 (nodes 3-14) SN – DMN / cEN	Interaction	-0.0269	0.2738	0.9894
V81	V81 (nodes 3-14) SN – DMN / cEN	Main effect	-0.0007	0.9660	0.9842
V83	V83 (nodes 5-14) cEN / DMN – DMN / cEN	Interaction	0.0337	0.1456	0.9894
V83	V83 (nodes 5-14) cEN / DMN – DMN / cEN	Main effect	-0.0168	0.3074	0.9615
V84	V84 (nodes 6-14) cEN / DMN – DMN / cEN	Interaction	-0.0020	0.9351	0.9894
V84	V84 (nodes 6-14) cEN / DMN – DMN / cEN	Main effect	-0.0047	0.7849	0.9615
V85	V85 (nodes 7-14) DMN / cEN – DMN / cEN	Interaction	-0.0099	0.6664	0.9894
V85	V85 (nodes 7-14) DMN / cEN – DMN / cEN	Main effect	0.0033	0.8320	0.9615
V87	V87 (nodes 9-14) cEN – DMN / cEN	Interaction	0.0509	0.0331	0.9894
V87	V87 (nodes 9-14) cEN – DMN / cEN	Main effect	-0.0181	0.2853	0.9615
V91	V91 (nodes 13-14) DMN – DMN / cEN	Interaction	-0.0003	0.9894	0.9894
V91	V91 (nodes 13-14) DMN – DMN / cEN	Main effect	-0.0045	0.7937	0.9615
V106	V106 (nodes 1-16) DMN – cEN	Interaction	0.0065	0.6916	0.9894
V106	V106 (nodes 1-16) DMN – cEN	Main effect	-0.0067	0.5607	0.9615
V108	V108 (nodes 3-16) SN – cEN	Interaction	0.0017	0.9311	0.9894
V108	V108 (nodes 3-16) SN – cEN	Main effect	-0.0160	0.2391	0.9615
V110	V110 (nodes 5-16) cEN / DMN – cEN	Interaction	0.0060	0.7369	0.9894
V110	V110 (nodes 5-16) cEN / DMN – cEN	Main effect	0.0026	0.8337	0.9615
V111	V111 (nodes 6-16) cEN / DMN – cEN	Interaction	-0.0159	0.3594	0.9894
V111	V111 (nodes 6-16) cEN / DMN – cEN	Main effect	0.0093	0.4492	0.9615
V112	V112 (nodes 7-16) DMN / cEN – cEN	Interaction	0.0117	0.5051	0.9894
V112	V112 (nodes 7-16) DMN / cEN – cEN	Main effect	0.0000	0.9969	0.9969
V114	V114 (nodes 9-16) cEN – cEN	Interaction	0.0069	0.7165	0.9894
V114	V114 (nodes 9-16) cEN – cEN	Main effect	0.0093	0.4846	0.9615
V118	V118 (nodes 13-16) DMN – cEN	Interaction	-0.0093	0.6828	0.9894
V118	V118 (nodes 13-16) DMN – cEN	Main effect	0.0064	0.6883	0.9615
V119	V119 (nodes 14 – 16) cEN – cEN	Interaction	-0.0003	0.9875	0.9894
V119	V119 (nodes 14 – 16) cEN – cEN	Main effect	0.0023	0.8799	0.9697
V172	V172 (nodes 1-20) DMN – DMN / cEN	Interaction	0.0212	0.2529	0.9894
V172	V172 (nodes 1-20) DMN – DMN / cEN	Main effect	-0.0082	0.5299	0.9615

Edge	Description	Effect	Estimate	P_value	P_value_FDR
V174	V174 (nodes 3-20) SN – DMN / CEN	Interaction	0.0155	0.4563	0.9894
V174	V174 (nodes 3-20) SN – DMN / CEN	Main effect	-0.0074	0.6139	0.9615
V176	V176 (nodes 5-20) cEN / DMN – DMN / cEN	Interaction	0.0122	0.5208	0.9894
V176	V176 (nodes 5-20) cEN / DMN – DMN / cEN	Main effect	-0.0058	0.6680	0.9615
V177	V177 (nodes 6-20) cEN – DMN / cEN	Interaction	-0.0106	0.5692	0.9894
V177	V177 (nodes 6-20) cEN – DMN / cEN	Main effect	0.0180	0.1765	0.9615
V178	V178 (nodes 7-20) DMN / cEN – DMN / cEN	Interaction	0.0043	0.8097	0.9894
V178	V178 (nodes 7-20) DMN / cEN – DMN / cEN	Main effect	-0.0046	0.7117	0.9615
V180	V180 (nodes 9-20) cEN – DMN / cEN	Interaction	-0.0021	0.9161	0.9894
V180	V180 (nodes 9-20) cEN – DMN / cEN	Main effect	-0.0056	0.6924	0.9615
V184	V184 (nodes 13-20) DMN – DMN / cEN	Interaction	-0.0046	0.8441	0.9894
V184	V184 (nodes 13-20) DMN – DMN / cEN	Main effect	-0.0016	0.9239	0.9783
V185	V185 (nodes 14-20) cEN – DMN / cEN	Interaction	-0.0332	0.1395	0.9894
V185	V185 (nodes 14-20) cEN – DMN / cEN	Main effect	0.0155	0.3304	0.9615
V191	V191 (nodes 1-21) DMN – cEN / DMN	Interaction	-0.0130	0.5335	0.9894
V191	V191 (nodes 1-21) DMN – cEN / DMN	Main effect	-0.0106	0.4684	0.9615
V193	V193 (nodes 3-21) SN – cEN / DMN	Interaction	-0.0201	0.3495	0.9894
V193	V193 (nodes 3-21) SN – cEN / DMN	Main effect	0.0023	0.8779	0.9697
V195	V195 (nodes 5-21) cEN / DMN – cEN / DMN	Interaction	0.0014	0.9420	0.9894
V195	V195 (nodes 5-21) cEN / DMN – cEN / DMN	Main effect	0.0028	0.8369	0.9615
V196	V196 (nodes 6-21) cEN – cEN / DMN	Interaction	0.0168	0.2887	0.9894
V196	V196 (nodes 6-21) cEN – cEN / DMN	Main effect	0.0084	0.4613	0.9615
V197	V197 (nodes 7-21) DMN / cEN – cEN / DMN	Interaction	0.0051	0.7995	0.9894
V197	V197 (nodes 7-21) DMN / cEN – cEN / DMN	Main effect	-0.0043	0.7627	0.9615
V199	V199 (nodes 9-21) cEN – cEN / DMN	Interaction	-0.0125	0.5346	0.9894
V199	V199 (nodes 9-21) cEN – cEN / DMN	Main effect	0.0087	0.5332	0.9615
V203	V203 (nodes 13-21) DMN – cEN / DMN	Interaction	0.0072	0.7450	0.9894
V203	V203 (nodes 13-21) DMN – cEN / DMN	Main effect	-0.0156	0.3140	0.9615
V204	V204 (nodes 14-21) cEN – cEN / DMN	Interaction	-0.0053	0.8282	0.9894
V204	V204 (nodes 14-21) cEN – cEN / DMN	Main effect	-0.0043	0.8085	0.9615
V206	V206 (nodes 16-21) cEN – cEN / DMN	Interaction	0.0144	0.4573	0.9894
V206	V206 (nodes 16-21) cEN – cEN / DMN	Main effect	0.0045	0.7445	0.9615
V210	V210 (nodes 20-21) DMN / cEN – cEN / DMN	Interaction	-0.0381	0.0755	0.9894
V210	V210 (nodes 20-21) DMN / cEN – cEN / DMN	Main effect	0.0153	0.3058	0.9615

STROBE Statement—checklist of items that should be included in reports of observational studies

	Item No.	Recommendation	Page No.	Relevant text from manuscript
**Title and abstract**	1	(*a*) Indicate the study's design with a commonly used term in the title or the abstract	1	
		(*b*) Provide in the abstract an informative and balanced summary of what was done and what was found	2	
**Introduction**				
Background/rationale	2	Explain the scientific background and rationale for the investigation being reported	4	
Objectives	3	State specific objectives, including any prespecified hypotheses	5	
**Methods**				
Study design	4	Present key elements of study design early in the paper	5	
Setting	5	Describe the setting, locations, and relevant dates, including periods of recruitment, exposure, follow-up, and data collection	5-7, SM	
Participants	6	(*a*) *Cohort study*—Give the eligibility criteria, and the sources and methods of selection of participants. Describe methods of follow-up<br>*Case-control study*—Give the eligibility criteria, and the sources and methods of case ascertainment and control selection. Give the rationale for the choice of cases and controls<br>*Cross-sectional study*—Give the eligibility criteria, and the sources and methods of selection of participants	5-9, SM	
		(*b*) *Cohort study*—For matched studies, give matching criteria and number of exposed and unexposed<br>*Case-control study*—For matched studies, give matching criteria and the number of controls per case	6-7, SM	

Variables	7	Clearly define all outcomes, exposures, predictors, potential confounders, and effect modifiers. Give diagnostic criteria, if applicable	6-9, SM
Data sources/ measurement	8*	For each variable of interest, give sources of data and details of methods of assessment (measurement). Describe comparability of assessment methods if there is more than one group	6-9, SM
Bias	9	Describe any efforts to address potential sources of bias	8-10, SM
Study size	10	Explain how the study size was arrived at	5-6, SM

Continued on next page

Quantitative variables	11	Explain how quantitative variables were handled in the analyses. If applicable, describe which groupings were chosen and why	10, SM
Statistical methods	12	(*a*) Describe all statistical methods, including those used to control for confounding	10, SM
		(*b*) Describe any methods used to examine subgroups and interactions	10, SM
		(*c*) Explain how missing data were addressed	10-11, SM
		(*d*) *Cohort study*—If applicable, explain how loss to follow-up was addressed<br>*Case-control study*—If applicable, explain how matching of cases and controls was addressed<br>*Cross-sectional study*—If applicable, describe analytical methods taking account of sampling strategy	NA
		(*e*) Describe any sensitivity analyses	10-11, SM
**Results**			
Participants	13*	(a) Report numbers of individuals at each stage of study—eg numbers potentially eligible, examined for eligibility, confirmed eligible, included in the study, completing follow-up, and analysed	SM Fig 1
		(b) Give reasons for non-participation at each stage	SM Fig 1
		(c) Consider use of a flow diagram	SM Fig 1
Descriptive data	14*	(a) Give characteristics of study participants (eg demographic, clinical, social) and information on exposures and potential confounders	11-12
		(b) Indicate number of participants with missing data for each variable of interest	11-15, SM
		(c) *Cohort study*—Summarise follow-up time (eg, average and total amount)	11, SM
Outcome data	15*	*Cohort study*—Report numbers of outcome events or summary measures over time	13-14, Fig 1B, SM
		Case-control study—Report numbers in each exposure category, or summary measures of exposure	

		Cross-sectional study—Report numbers of outcome events or summary measures	
Main results	16	(*a*) Give unadjusted estimates and, if applicable, confounder-adjusted estimates and their precision (eg, 95% confidence interval). Make clear which confounders were adjusted for and why they were included	11-16, SM
		(*b*) Report category boundaries when continuous variables were categorized	9-10, SM (confounds)
		(*c*) If relevant, consider translating estimates of relative risk into absolute risk for a meaningful time period	

Continued on next page

Other analyses	17	Report other analyses done—eg analyses of subgroups and interactions, and sensitivity analyses	14-17
**Discussion**			
Key results	18	Summarise key results with reference to study objectives	13-15
Limitations	19	Discuss limitations of the study, taking into account sources of potential bias or imprecision. Discuss both direction and magnitude of any potential bias	19-21
Interpretation	20	Give a cautious overall interpretation of results considering objectives, limitations, multiplicity of analyses, results from similar studies, and other relevant evidence	18-19
Generalisability	21	Discuss the generalisability (external validity) of the study results	21-22
**Other information**			
Funding	22	Give the source of funding and the role of the funders for the present study and, if applicable, for the original study on which the present article is based	24
